# Near-critical SIR epidemic on a random graph with given degrees

**DOI:** 10.1007/s00285-016-1043-z

**Published:** 2016-07-30

**Authors:** Svante Janson, Malwina Luczak, Peter Windridge, Thomas House

**Affiliations:** 1 Department of Mathematics, Uppsala University, PO Box 480, 751 06 Uppsala, Sweden; 2 School of Mathematical Sciences, Queen Mary University of London, Mile End Road, London, E1 4NS UK; 3 School of Mathematics, University of Manchester, Manchester, M13 9PL UK

**Keywords:** SIR epidemic, Random graph with given degrees, Configuration model, Critical window, 05C80, 60F99, 60J28, 92D30

## Abstract

Emergence of new diseases and elimination of existing diseases is a key public health issue. In mathematical models of epidemics, such phenomena involve the process of infections and recoveries passing through a critical threshold where the basic reproductive ratio is 1. In this paper, we study near-critical behaviour in the context of a susceptible-infective-recovered epidemic on a random (multi)graph on *n* vertices with a given degree sequence. We concentrate on the regime just above the threshold for the emergence of a large epidemic, where the basic reproductive ratio is $1 + \omega (n) n^{-1/3}$, with $\omega (n)$ tending to infinity slowly as the population size, *n*, tends to infinity. We determine the probability that a large epidemic occurs, and the size of a large epidemic. Our results require basic regularity conditions on the degree sequences, and the assumption that the third moment of the degree of a random susceptible vertex stays uniformly bounded as $n \rightarrow \infty$. As a corollary, we determine the probability and size of a large near-critical epidemic on a standard binomial random graph in the ‘sparse’ regime, where the average degree is constant. As a further consequence of our method, we obtain an improved result on the size of the giant component in a random graph with given degrees just above the critical window, proving a conjecture by Janson and Luczak.

## Introduction

Infectious diseases continue to pose a serious threat to individual and public health. Accordingly, health organisations are constantly seeking to analyse and assess events that may present new challenges. These may include acts of bioterrorism, and other events indicating emergence of new infections, which threaten to spread rapidly across the globe facilitated by the efficiency of modern transportation. Likewise, a lot of effort is being directed into suppressing outbreaks of established diseases such as influenza and measles, as well as into eliminating certain endemic diseases, such as polio and rabies.

In an SIR epidemic model, an infectious disease spreads through a population where each individual is either susceptible, infective or recovered. The population is represented by a network (graph) of contacts, where the vertices of the network correspond to individuals and the edges correspond to potential infectious contacts. Different individuals will have different lifestyles and patterns of activity, leading to different numbers of contacts; for simplicity, we assume that each person’s contacts are randomly chosen from among the rest of the population. The degree of a vertex is the number of contacts of the corresponding individual.

We assume that infectious individuals become recovered at rate $\rho \geqslant 0$ and infect each neighbour at rate $\beta > 0$. Then the basic reproductive ratio ${\mathcal R}_0$ (i.e. the average number of secondary cases of infection arising from a single case) is given by the average size-biased susceptible degree times the probability that a given infectious contact takes place before the infective individual recovers.

Emergence and elimination of a disease involves the process of infectious transitions and recoveries being pushed across a critical threshold, usually corresponding to the basic reproductive ratio ${\mathcal R}_0$ equal to 1, see Antia et al. (2003), Bull and Dykhuizen (2003), O’Regan and Drake (2013) and Scheffer et al. (2009). For example, a pathogen mutation can increase the transmission rate and make a previously ‘subcritical’ disease (i.e. not infectious enough to cause a large outbreak) into a ‘supercritical’ one, where a large outbreak may occur, see Antia et al. (2003). Moreover, after a major outbreak in the supercritical case, disease in the surviving population is subcritical. However, subsequently, as people die and new individuals are born (i.e. immunity wanes), ${\mathcal R}_0$ will slowly increase, and, when it passes 1, another major outbreak may occur. Equally, efforts at disease control may result in subcriticality for a time, but then inattention may lead to an unnoticed parameter shift to supercriticality. Thus, under certain conditions, one can expect most large outbreaks to occur close to criticality, and so there is practical interest in theoretical understanding of the behaviour of near-critical epidemics.

Critical SIR epidemics have been studied for populations with complete mixing, under different assumptions, by Ben-Naim and Krapivsky (2004), Gordillo et al. (2008), Hofstad et al. (2010) and Martin-Löf (1998); this is equivalent to studying epidemic processes on the complete graph, or on the Erdős-Rényi graph *G*(*n*, *p*). In Ben-Naim and Krapivsky (2004), near-criticality is discussed using non-rigorous arguments. Martin-Löf (1998) studies a generalized Reed–Frost epidemic model, where the number of individuals that a given infective person infects has an essentially arbitrary distribution. The binomial case is equivalent to studying the random graph *G*(*n*, *p*) on *n* vertices with edge probability *p*. The author considers the regime where ${\mathcal R}_0 - 1 = a n^{-1/3}$ and the initial number of infectives is $b n^{1/3}$, for constant *a*, *b*. A limit distribution is derived for the final size of the epidemic, observing bimodality for certain values of *a* and *b* (corresponding to ‘small’ and ‘large’ epidemics). Further analytical properties of the limit distribution are derived in Hofstad et al. (2010). In Gordillo et al. (2008), a standard SIR epidemic for populations with homogeneous mixing is studied, with vaccinations during the epidemic; a diffusion limit is derived for the final size of a near-critical epidemic.

In the present paper, we address near-critical phenomena in the context of an epidemic spreading in a population of a large size *n*, where the underlying graph (network) is a random (multi)graph with given vertex degrees. In other words, we specify the number of contacts for each individual, and consider a graph chosen uniformly at random from among all graphs with the specified sequence of contact numbers. This random graph model allows for greater inhomogeneity, with a rather arbitrary distribution of the number of contacts for different persons. We study the regime just above the critical threshold for the emergence of a large epidemic, where the basic reproductive ratio is $1 + \omega (n) n^{-1/3}$, with $\omega (n)$ growing large as the population size *n* grows. (For example, when the population size is about 1 million, we could consider ${\mathcal R}_0$ of order about 1.01.)

From the theory of branching processes, at the start of an epidemic, each infective individual leads to a large outbreak with probability of the order ${\mathcal R}_0-1$. Roughly, our results confirm the following, intuitively clear from the above observation, picture. If the size *n* of the population is very large, with the initial total infectious degree $X_{\mathrm {I},0}$ (i.e. total number of potential infectious contacts at the beginning of the epidemic or total number of acquaintances of initially infectious individuals) much larger than $({\mathcal R}_0-1)^{-1}$, then a large epidemic will occur with high probability. If the initial total infectious degree is much smaller than $({\mathcal R}_0-1)^{-1}$, then the outbreak will be contained with high probability. In the intermediate case where $X_{\mathrm {I},0}$ and $({\mathcal R}_0-1)^{-1}$ are of the same order of magnitude, a large epidemic can occur with positive probability, of the order $\exp \bigl (-c X_{\mathrm {I},0} ({\mathcal R}_0-1)\bigr )$, for some positive constant *c*. So, if the population size is about a million, and ${\mathcal R}_0$ about 1.01, then $X_{\mathrm {I},0}$ much larger than 100 will result in a large epidemic with high probability. On the other hand, if $X_{\mathrm {I},0}$ is less than 10, say, than the outbreak will be contained with high probability.

Furthermore, we determine the likely size of a large epidemic. Here, there are three possible regimes, depending on the size of the initial total infectious degree relative to $n ({\mathcal R}_0-1)^2$. Broadly speaking, if $X_{\mathrm {I},0}$ is much larger than $n ({\mathcal R}_0-1)^2$, then the total number of people infected will be proportional to $(n X_{\mathrm {I},0})^{1/2}$. On the other hand, if $X_{\mathrm {I},0}$ is much smaller than $n ({\mathcal R}_0-1)^2$ then, in the event that there is a large epidemic, the total number of people infected will be proportional to $n ({\mathcal R}_0-1)$. The intermediate case where $X_{\mathrm {I},0}$ and $n ({\mathcal R}_0-1)^2$ are of the same order ‘connects’ the two extremal cases.

Note that, if $X_{\mathrm {I},0}$ is of the same or larger order of magnitude than $n ({\mathcal R}_0-1)^2$ (the first and third case in the paragraph above), then $X_{\mathrm {I},0} ({\mathcal R}_0-1)$ is very large, so a large epidemic does occur with high probability. This follows since, by our assumption, $n({\mathcal R}_0-1)^3 = \omega (n)^3$ is large for large *n*.

The above results are proven under fairly mild regularity assumptions on the shape of the degree distribution. We allow a non-negligible proportion of the population to be initally recovered, i.e. immune to the disease. (This also allows for the possibility that a part, not necessarily random, of the population is vaccinated before the outbreak, since the vaccinated individuals can be regarded as recovered.) We require that the third moment of the susceptible degree be bounded; in particular, that implies that the maximum susceptible degree in a population of size *n* is of the order no larger than $n^{1/3}$. So in particular, in a population of size 1 million, the super-spreaders (i.e. individuals with largest numbers of contacts) should not be able to infect more than around 100 individuals.

**Fig. 1:**
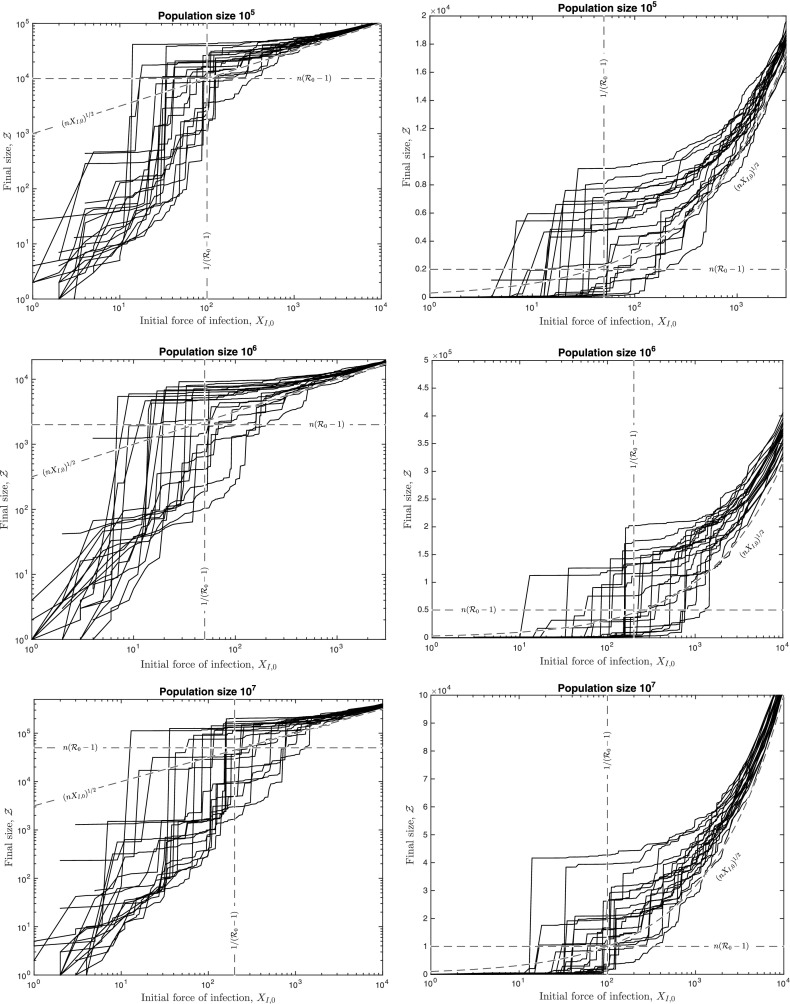
The relationship between epidemic final size and initial force of infection for 20 realisations of the network Sellke construction on a network with Poisson degree distribution with mean $\lambda$. Parameter sets are: $n=10^5, \lambda = 2.04, R_{0}=1.02$ (*top*); $n=10^6, \lambda = 2.02, R_{0}=1.01$ (*middle*); $n=10^7, \lambda = 2.01, R_{0}=1.005$ (*bottom*); and $\rho =1$ throughout (note that these parameter choices imply that $\beta =1$)

To demonstrate this behaviour for a particular example, we used stochastic simulations that make use of special Monte Carlo techniques that allow us to consider multiple initial conditions within the same realisation of the process. The algorithm is described in Appendix A. Figure 1 shows our results for the relationship between the epidemic final size $\mathcal {Z}$ and the initial force of infection $X_{I,0}$ for 20 realisations of the process, with each realisation involving multiple different initial conditions. The model rate parameters are $\rho =1$ and $\beta =1$. The network has Poisson degree distribution with mean $\lambda$ and was generated as an Erdős–Rényi random graph with edge probability $\lambda /n$. We implement scaling at different population sizes giving parameter sets $(n = 10^{5}, \lambda = 2.04, R_{0} = 1.02), (n = 10^{6}, \lambda = 2.02, R_{0} = 1.01)$ and $(n = 10^{7}, \lambda = 2.01, R_{0} = 1.005)$. These plots show the emergence of the three epidemic sizes that our results predict as *n* increases, i.e. ‘small’ epidemics of size *O*(1), ‘large’ epidemics of size proportional to $(nX_{I,0})^{1/2}$, and ‘large’ epidemics of size comparable to $n({\mathcal R}_0-1)$.

Epidemics on graphs with given degrees have been considered in a number of recent studies, both within the mathematical biology and probability communities. A set of ordinary differential equations approximating the time evolution of a large epidemic were obtained by Volz (2008), see also Miller (2011), and also Miller et al. (2012). These papers consider the case where the epidemic starts very small. Differential equations for an epidemic starting with a large number of infectives appear in Miller (2014). Convergence of the random process to these equations in the case where the second moment of the degree of a random vertex is uniformly bounded (both starting with only few infectives and with a large number of infectives) was proven in Janson et al. (2014). (See also Decreusefond et al. 2012; Bohman and Picollelli 2012, where related results are proven in the case where the fifth moment of the degree of a random vertex is uniformly bounded and in the case of bounded vertex degrees respectively. See also Barbour and Reinert 2013 for results in the case of bounded vertex degrees and general infection time distributions.)

However, we appear to be the first to study the ‘barely supercritical’ SIR epidemic on a random graph with given degrees. As a corollary, we determine the probability and size of a large near-critical epidemic on a sparse binomial (Erdős–Rényi) random graph, also to our knowledge the first such results in the literature.

Our approach also enables us to prove the conjecture of Janson and Luczak (2009), establishing their Theorem 2.4 concerning the size of the largest component in the barely supercritical random graph with given vertex degrees under weakened assumptions.

We proceed in the spirit of Janson et al. (2014) and Janson and Luczak (2009), evolving the epidemic process simultaneously with constructing the random multigraph. The main technical difficulties involve delicate concentration of measure estimates for quantities of interest, such as the current total degrees of susceptible, recovered and infective vertices. Also, our proofs involve couplings of the evolution of the total infective degree with suitable Brownian motions.

The remainder of the paper is organised as follows. In Sect. 2, we define our notation and state our main results (Theorems 2.4, 2.5). Section 3 is devoted to the proof of Theorem 2.4; to this end, we define a time-changed version of the epidemic and use the modified process to prove concentration of measure estimates for various quantities of interest. In Sect. 4, we prove Theorem 2.5. In Appendix B, we state and prove a new result concerning the size of the giant component in the supercritical random (multi)graph with a given degree sequence.

## Model, notation, assumptions and results

Let $n \in \mathbb {N}$ and let $(d_i)_{i = 1}^n= (d_i^{(n)})_{i = 1}^n$ be a given sequence of non-negative integers. Let $G= G(n, (d_i)_{i = 1}^n )$ be a simple graph (no loops or multiple edges) with *n* vertices, chosen uniformly at random subject to vertex *i* having degree $d_i$ for $i=1, \ldots , n$, tacitly assuming there is any such graph at all ($\sum _{i = 1}^n d_i$ must be even, at least). For each $k \in {\mathbb Z}^+$, let $n_k$ denote the total number of vertices with degree *k*.

Given the graph $G$, the epidemic evolves as a continuous-time Markov chain. Each vertex is either susceptible, infective or recovered. Every infective vertex recovers at rate $\rho _n \geqslant 0$ and also infects each susceptible neighbour at rate $\beta _n > 0$.

Let $n_{\mathrm {S}}$, $n_{\mathrm {I}}$, and $n_{\mathrm {R}}$ denote the initial numbers of susceptible, infective and recovered vertices, respectively. Further, let $n_{\mathrm {S},k}$, $n_{\mathrm {I},k}$ and $n_{\mathrm {R},k}$ respectively, be the number of these vertices with degree $k \geqslant 0$. Thus, $n_{\mathrm {S}}+ n_{\mathrm {I}}+ n_{\mathrm {R}}= n$ and $n_{\mathrm {S}}= \sum _{k=0}^\infty n_{\mathrm {S},k}$, $n_{\mathrm {I}}= \sum _{k=0}^\infty n_{\mathrm {I},k}$, $n_{\mathrm {R}}= \sum _{k=0}^\infty n_{\mathrm {R},k}$, and $n_{k}= n_{\mathrm {S},k}+ n_{\mathrm {I},k}+ n_{\mathrm {R},k}$. We assume that this information is given with the degree sequence. Note that all these quantities (as well as many of the quantities introduced below) depend on *n*. To lighten the notation, we usually do not indicate the *n* dependence explicitly.

### Remark 2.1

We allow $n_{\mathrm {R}}>0$, i.e., that some vertices are “recovered” (i.e., immune) already when we start. It is often natural to take $n_{\mathrm {R}}=0$, but one application of $n_{\mathrm {R}}>0$ is to study the effect of vaccination; this was done in a related situation in Janson et al. (2014) and we leave the corresponding corollaries of the results below to the reader. Note that initially recovered vertices are not themselves affected by the epidemic, but they influence the structure of the graph and thus the course of the epidemic, so we cannot just ignore them.

### Remark 2.2

Note that initially susceptible, infective and recovered vertices can have different degree distributions. However, we assume that given the vertex degrees, the connections in the graph are made at random, independently of the initial status of the vertices. Equivalently, if we first construct the connections at random, we assume that the initially infective and recovered vertices are selected at random, where we may select on the basis of their degrees, but not on any other properties of the graph.

For example, if some individuals are vaccinated before the outbreak, and thus are regarded as initially recovered as discussed in Remark 2.1, then our model covers the case when the vaccinated individuals are chosen uniformly at random, as well as the case when vaccination is directed at high-risk groups and individuals are vaccinated with a probability depending on their degree (number of contacts), but the model does not include more complicated vaccination schemes that take into account also, e.g., the degrees of the contacts.

Similarly, if the disease has been spreading for some time before we start our calculations, then there are correlations because an infected vertex that was not initially infected has to be connected to an infected or recovered vertex (the one that infected it); thus our model is not directly applicable. As suggested by an anonymous referee, if we know the history so far of the epidemic, this can be handled be removing those edges that have tried to infect (whether to a susceptible individual or not); the remaining network is uniformly random with given vertex degrees and our model applies to it.

The basic reproductive ratio ${\mathcal R}_0$ is commonly used in the context of epidemic models, and defines the average number of new cases created by a case of infection. In analogy with the limiting case in Janson et al. (2014, (2.23)), for the SIR epidemic on a random graph with a given degree sequence, we define

2.1 $$\begin{aligned} {\mathcal R}_0 = {\mathcal R}_0^{(n)} := \frac{\beta _n}{\rho _n + \beta _n} \frac{\sum _{k=0}^{\infty } (k-1)kn_{\mathrm {S},k}}{\sum _{k=0}^{\infty } k n_k}. \end{aligned}$$

Here, the probability that an infective half-edge infects another half-edge before recovering is $\frac{\beta _n}{\rho _n + \beta _n}$, and the average increase in the number of infective half-edges due to such an infection event is $\frac{\sum _{k=0}^{\infty } (k-1)kn_{\mathrm {S},k}}{\sum _{k=0}^{\infty } k n_k}$, and these are approximately independent of one another, and approximately independent for different half-edges.

Note that the basic reproductive ratio ${\mathcal R}_0$ determines the approximate geometric growth rate of the disease during the early stages of the epidemic. The value ${\mathcal R}_0 =1$ is therefore the threshold for the epidemic to take off in the population, in the sense that, if ${\mathcal R}_0 > 1$, then a macroscopic fraction of the susceptibles can be infected (Andersson 1999; Newman 2002; Volz 2008; Bohman and Picollelli 2012; Janson et al. 2014). Here we will consider the case where ${\mathcal R}_0 = 1 + \omega (n) n^{-1/3}$, with $\omega (n)$ tending to infinity slowly (slower than $n^{1/3}$) as $n \rightarrow \infty$.

It turns out that, rather than working with the quantity ${\mathcal R}_0-1$, it is easier to work with a quantity $\alpha _n$ defined by

2.2 $$\begin{aligned} \alpha _n {:=} - (1 + \rho _n/\beta _n)\sum _{k=0}^\infty k n_{k}/ n_{\mathrm {S}}+ \sum _{k=0}^\infty k(k-1)n_{\mathrm {S},k}/n_{\mathrm {S}}. \end{aligned}$$

Note that

2.3 $$\begin{aligned} \alpha _n = ({\mathcal R}_0-1) \frac{\rho _n + \beta _n}{\beta _n} \frac{\sum _{k=0}^{\infty } k n_k}{n_{\mathrm {S}}}. \end{aligned}$$

Our assumptions below imply that $1\leqslant \frac{\rho _n + \beta _n}{\beta _n} =O(1)$ and that $\frac{\sum _{k=0}^{\infty } k n_k}{n_{\mathrm {S}}}$ is bounded and bounded away from 0 as $n \rightarrow \infty$, see Remark 2.9. Hence $({\mathcal R}_0-1)\alpha _n^{-1}$ is bounded and bounded away from 0, and so $\alpha _n$ is equivalent to ${\mathcal R}_0-1$ as a measure of distance from criticality; see further (2.22). In particular, we could rephrase our assumptions and results in terms of ${\mathcal R}_0-1$ instead of $\alpha _n$, but it seems that the mathematics works out more cleanly using $\alpha _n$. Also, we can expect an initial growth if and only if $\alpha _n>0$.

We consider asymptotics as $n \rightarrow \infty$, and all unspecified limits below are as $n \rightarrow \infty$. Throughout the paper we use the notation $o_{\mathrm p}$ in a standard way, as in Janson (2011). That is, for a sequence of random variables $(Y^{(n)})_1^\infty$ and real numbers $(a_n)_1^\infty$, ‘$Y^{(n)}= o_p(a_n)$’ means $Y^{(n)}/a_n \overset{\mathrm {p}}{\longrightarrow }0$. Similarly, $Y^{(n)}=O_{\mathrm p}(1)$ means that, for every $\varepsilon >0$, there exists $K_\varepsilon$ such that $\mathbb {P}{}(|Y^{(n)}|>K_\varepsilon )<\varepsilon$ for all *n*. Given a sequence of events $(A_n)_1^\infty$, $A_n$ is said to hold w.h.p. (with high probability) if $\mathbb {P}{}(A_n) \rightarrow 1$.

Our assumptions are as follows. (See also the remarks below.) Let $D_{\mathrm {S},n}$ denote the degree of a randomly chosen susceptible vertex, so $\mathbb {P}(D_{\mathrm {S},n}= k) = n_{\mathrm {S},k}/n_{\mathrm {S}}$ for each $k \geqslant 0$.

1. $D_{\mathrm {S},n}$ converges in distribution to a probability distribution $(p_k)_{k = 0}^\infty$ with a finite and positive mean $\lambda :=\sum _{k=0}^\infty kp_k$, i.e.
  2.4 $$\begin{aligned} \frac{n_{\mathrm {S},k}}{n_{\mathrm {S}}} \rightarrow p_k, \quad k \geqslant 0. \end{aligned}$$
2. The third power $D_{\mathrm {S},n}$ is uniformly integrable as $n\rightarrow \infty$. That is, given $\varepsilon > 0$, there exists $M > 0$ such that, for all *n*,
  2.5 $$\begin{aligned} \sum _{k > M}\frac{k^3n_{\mathrm {S},k}}{n_{\mathrm {S}}} < \varepsilon . \end{aligned}$$
3. The second moment of the degree of a randomly chosen vertex is uniformly bounded, i.e. $\sum _{k=0}^\infty k^2n_{k}= O(n)$.
4. As $$n \rightarrow \infty $$,
  2.6 $$\begin{aligned} \alpha _n \rightarrow 0 \quad \mathrm {and} \quad n_{\mathrm {S}}\alpha _n^3 \rightarrow \infty . \end{aligned}$$
5. The total degree $\sum _{k=0}^\infty kn_{\mathrm {I},k}$ of initially infective vertices satisfies
  2.7 $$\begin{aligned} \sum _{k=0}^\infty kn_{\mathrm {I},k}=o(n), \end{aligned}$$
  and the limit
  2.8 $$\begin{aligned} \nu {:=} \lim _{n \rightarrow \infty }\frac{1}{n_{\mathrm {S}}\alpha _n^2}\sum _{k=0}^\infty kn_{\mathrm {I},k}\in [0,\infty ] \end{aligned}$$
  exists (but may be 0 or $\infty$). Furthermore, either $\nu =0$ or
  2.9 $$\begin{aligned} d_{\mathrm {I},*}:=\max \{k:n_{\mathrm {I},k}\geqslant 1\}=o\left( \sum _{k=0}^{\infty }kn_{\mathrm{I},k}\right) . \end{aligned}$$
6. We have $$p_0 + p_1 + p_2 < 1$$.
7. $$\liminf _{{n\rightarrow \infty }} n_S/n>0$$.

We will repeatedly use the fact that (D2) implies that there exists a constant $c_0$ such that, for all *n*,

2.10 $$\begin{aligned} \sum _{k=0}^\infty k^3 n_{\mathrm {S},k}= n_{\mathrm {S}}\mathbb {E}{}D_{\mathrm {S},n}^3 \leqslant c_0 n. \end{aligned}$$

### Remark 2.3

Assumption (D1) says $D_{\mathrm {S},n}\overset{\mathrm {d}}{\longrightarrow }D_{\mathrm {S}}$, where $D_{\mathrm {S}}$ has distribution $(p_k)_{k = 0}^\infty$. Given (D1), assumption (D2) is equivalent to $\mathbb {E}{}D_{\mathrm {S},n}^3 \rightarrow \mathbb {E}{}D_{\mathrm {S}}^3 < \infty$. Furthermore, (D2) implies uniform integrability of $D_{\mathrm {S},n}$ and $D_{\mathrm {S},n}^2$, so $\mathbb {E}{}D_{\mathrm {S},n}\rightarrow \mathbb {E}{}D_{\mathrm {S}}$ and $\mathbb {E}{}D_{\mathrm {S},n}^2 \rightarrow \mathbb {E}{}D_{\mathrm {S}}^2$. Assumptions (D2) and (D7) further imply that

2.11 $$\begin{aligned} d_{\mathrm {S},*}:=\max \{k:n_{\mathrm {S},k}\geqslant 1\}=o(n_{\mathrm {S}}^{1/3}). \end{aligned}$$

Using the notation in Remark 2.3, $\lambda =\mathbb {E}{}D_{\mathrm {S}}$. Furthermore, let

2.12 $$\begin{aligned} \lambda _2&:= \sum _{k=0}^\infty k(k-1)p_k=\mathbb {E}{}D_{\mathrm {S}}(D_{\mathrm {S}}-1), \end{aligned}$$

2.13 $$\begin{aligned} \lambda _3&:= \sum _{k=0}^\infty k(k-1)(k-2)p_k= \mathbb {E}{}D_{\mathrm {S}}(D_{\mathrm {S}}-1)(D_{\mathrm {S}}-2). \end{aligned}$$

Then, the uniform integrability (D2) of $D_{\mathrm {S},n}^3$ implies $\lambda _2,\lambda _3<\infty$ and furthermore

2.14 $$\begin{aligned} \lambda _2&= \lim _{n\rightarrow \infty }\mathbb {E}{}D_{\mathrm {S},n}(D_{\mathrm {S},n}-1) =\lim _{n\rightarrow \infty }\sum _{k=0}^\infty k(k-1)\frac{n_{\mathrm {S},k}}{n_{\mathrm {S}}}, \end{aligned}$$

2.15 $$\begin{aligned} \lambda _3&= \lim _{n\rightarrow \infty }\mathbb {E}{}D_{\mathrm {S},n}(D_{\mathrm {S},n}-1)(D_{\mathrm {S},n}-2) =\lim _{n\rightarrow \infty }\sum _{k=0}^\infty k(k-1)(k-2)\frac{n_{\mathrm {S},k}}{n_{\mathrm {S}}} \end{aligned}$$

Also, $$\lambda ,\lambda _2,\lambda _3> 0$$ by (D6).

Let $G^*=G^* (n, (d_i)_1^n )$ be the random multigraph with given degree sequence $(d_i)_1^n$ defined by the *configuration model*: we take a set of $d_i$ half-edges for each vertex *i* and combine half-edges into edges by a uniformly random matching (see e.g. Bollobás 2001). Conditioned on the multigraph being simple, we obtain $G = G (n, (d_i)_1^n )$, the uniformly distributed random graph with degree sequence $(d_i)_1^n$. The configuration model has been used in the study of epidemics in a number of earlier works, see, for example, Andersson (1998), Ball and Neal (2008), Britton et al. (2007), Decreusefond et al. (2012), Bohman and Picollelli (2012). As in many other papers, including Janson et al. (2014), we prove our results for the SIR epidemic on $G^*$, and, by conditioning on $G^*$ being simple, we then deduce that these results also hold for the SIR epidemic on $G$. The results below thus hold for both the random multigraph $G^*$ and the random simple graph $G$.

This argument relies on the probability that $G^*$ is simple being bounded away from zero as $n \rightarrow \infty$; by the main theorem of Janson (2009b) (see also Janson 2014) this occurs provided condition (D3) holds. Most of the results below are of the “w.h.p.” type (or can be expressed in this form); then this transfer to the simple graph case is routine and will not be commented on further. The exception is Theorem 2.5(iii), where we obtain a limiting probability strictly between 0 and 1, and we therefore need a more complicated argument, see Sect. 4; we also use an extra assumption in this case.

We now state our main result, that, under the conditions above, the epidemic is either very small, or of a size at least approximatively proportional to $n\alpha _n$ (and thus to $n({\mathcal R}_0-1)$). As just said, the theorem holds for both the multigraph $G^*$ and the simple graph $G$.

### Theorem 2.4

Suppose that (D1)–(D7) hold.

Let ${\mathcal Z}$ be the total number of susceptible vertices that ever get infected.

(i) If $\nu =0$, then there exists a sequence $\varepsilon _n\rightarrow 0$ such that, for each *n*, w.h.p. one of the following holds.
  - ${\mathcal Z}/n_{\mathrm {S}}\alpha _n < \varepsilon _n$ (the epidemic is small and ends prematurely).
  - $|{\mathcal Z}/n_{\mathrm {S}}\alpha _n -2\lambda /\lambda _3| < \varepsilon _n$ (the epidemic is large and its size is well concentrated).
(ii) If $0<\nu <\infty$, then ${\mathcal Z}/n_{\mathrm {S}}\alpha _n \overset{\mathrm {p}}{\longrightarrow }\lambda (1 + \sqrt{1+2\nu \lambda _3})/\lambda _3$.
(iii) If $\nu =\infty$, then
  2.16 $$\begin{aligned} \frac{{\mathcal Z}}{\bigl (n_{\mathrm {S}}\sum _{k=0}^\infty kn_{\mathrm {I},k}\bigr )^{1/2}} \overset{\mathrm {p}}{\longrightarrow }\frac{\sqrt{2}\,\lambda }{\sqrt{\lambda _3}} \end{aligned}$$

Moreover, in cases (i)(b), (ii) and (iii), the following holds. Let ${\mathcal Z}_k$ be the number of degree $k\geqslant 0$ susceptible vertices that ever get infected. Then

2.17 $$\begin{aligned} \sum _{k=0}^\infty \biggl |\frac{{\mathcal Z}_k}{{\mathcal Z}}&- \frac{kp_k}{\lambda } \biggr | \overset{\mathrm {p}}{\longrightarrow }0. \end{aligned}$$

Thus, (2.17) says that, except in the case (i)(a), the total variation distance between the degree distribution $({\mathcal Z}_k/{\mathcal Z})$ of the vertices that get infected and the size-biased distribution $(kp_k/\lambda )$ converges to 0 in probability.

Note that case (i) of Theorem 2.4 says that, for a range of initial values of the number of infective half-edges (viz. when $\nu =0$), if the epidemic takes off at all, then it has approximately the size $(2\lambda /\lambda _3)n_{\mathrm {S}}\alpha _n$. Hence, in this range, the size of the epidemic does (to the first order) not depend on the initial number of infective half-edges (only the probability of a large outbreak does), so this can be seen as the “natural” size of an epidemic. This also means that in this range, most of the outbreak can be traced back to a single initial infective half-edge.

However, when the initial number of infective half-edges number gets larger, the many small outbreaks coming from the different initially infective half-edges will add up to a substantial outbreak. So there is a threshold where this bulk of combined small outbreaks is of about the same size as the “natural” size of a large outbreak. The value $\nu$ is, in the limit as $n \rightarrow \infty$, the ratio of the initial number divided by this threshold, so it shows, roughly, whether the combined small outbreaks give a large contribution to the outbreak or not. Our theorem then shows that, if the initial number of infective half-edges is larger (to be precise, $\nu >0$), then they force a larger outbreak, with a size that is proportional to the square root of the initial number of infective half-edges in the range $\nu =\infty$. (For $0<\nu <\infty$, there is a smooth transition between the two extremal cases.)

The following result gives conditions for the occurrence of a large epidemic in Theorem 2.4(i). In anticipation of later notation, let $X_{\mathrm {I},0}:= \sum _{k=0}^\infty k n_{\mathrm {I},k}$ be the total degree of initially infective vertices (i.e. the total number of initially infective half-edges).

### Theorem 2.5

Suppose that the assumptions of Theorem 2.4 are satisfied with $\nu =0$.

(i) If $\alpha _n X_{\mathrm {I},0} \rightarrow 0$, then ${\mathcal Z}= o_{\mathrm p}(\alpha _n^{-2})= o_{\mathrm p}(n_{\mathrm {S}}\alpha _n)$, and thus case (i)(a) in Theorem 2.4 occurs w.h.p.
(ii) If $\alpha _n X_{\mathrm {I},0} \rightarrow \infty$ then case (i)(b) in Theorem 2.4 occurs w.h.p.
(iii) Suppose that $\alpha _n X_{\mathrm {I},0}$ is bounded above and below. In the simple graph case, assume also that $\sum _{k\geqslant 1} k^2 n_{\mathrm {I},k}=o(n)$ and $\sum _{k\geqslant \alpha _n^{-1}} k^2 n_{\mathrm {R},k}=o(n)$. Then both cases (i)(a) and (i)(b) in Theorem 2.4 occur with probabilities bounded away from 0 and 1. Furthermore, if $d_{\mathrm {I},*}=o(X_{\mathrm {I},0})$, then the probability that case (i)(a) in Theorem 2.4 occurs is
  2.18 $$\begin{aligned} \exp \left( -\frac{\lambda _2+\lambda + \sum _{k=0}^\infty k n_{\mathrm {R},k}/n_{\mathrm {S}}}{\lambda _2\lambda _3} \alpha _n X_{\mathrm {I},0}\right) +o(1). \end{aligned}$$
  Moreover, in the case the epidemic is small, ${\mathcal Z}=O_{\mathrm p}\bigl (\alpha _n^{-2}\bigr )$.

Note that $\sum _{k=0}^\infty k n_{\mathrm {R},k}/n_{\mathrm {S}}$ in (2.18) is bounded because of (D3) and (D7), and that (2.18) holds in cases (i) and (ii) too. A more complicated formula extending (2.18) holds also in the case when the condition $d_{\mathrm {I},*}=o(X_{\mathrm {I},0})$ fails, see (4.63) in Remark 4.4.

### Remark 2.6

The quantity $\nu \geqslant 0$ controls the initial number of infective contacts. If $\nu > 0$, so a large epidemic occurs by Theorem 2.4, then

$$\begin{aligned} \alpha _nX_{\mathrm {I},0}= \alpha _n \sum _{k=0}^\infty k n_{\mathrm {I},k}= (n_{\mathrm {S}}\alpha _n^3) \frac{\sum _{k=0}^\infty k n_{\mathrm {I},k}}{n_{\mathrm {S}}\alpha _n^2} \rightarrow \infty , \end{aligned}$$

by (2.6) and (2.8); hence the condition in Theorem 2.5(ii) holds automatically when $\nu >0$.

### Remark 2.7

The condition (2.7) that the total degree of initally infective vertices is *o*(*n*) is, by (D3) and the Cauchy–Schwarz inequality, equivalent to $n_I=o(n)$, at least if we ignore isolated infective vertices. Note that the opposite case, when $n_I/n$ has a strictly positive limit, is treated in Janson et al. (2014, Theorems 2.6 and 2.7) (under otherwise similar assumptions).

### Remark 2.8

The assumption (2.9) (which is required only when $\nu >0$) says that no single infective vertex has a significant fraction of the total infective degree.

### Remark 2.9

Assuming (D1) and (D2), the assumption $\alpha _n\rightarrow 0$ in (D4) is equivalent to ${\mathcal R}_0\rightarrow 1$, as said above. To see this, note that (D1) and (D2) imply (see Remark 2.3)

2.19 $$\begin{aligned} \sum _{k=0}^\infty k n_k/n_{\mathrm {S}}\geqslant \sum _{k=0}^\infty kn_{\mathrm {S},k}/n_{\mathrm {S}}= \mathbb {E}{}D_{\mathrm {S},n}\rightarrow \mathbb {E}{}D_{\mathrm {S}}=\lambda >0. \end{aligned}$$

If $\alpha _n\rightarrow 0$, then (2.19) and (2.3) imply that ${\mathcal R}_0\rightarrow 1$.

Conversely, still assuming (D1) and (D2), if ${\mathcal R}_0\rightarrow 1$, then it follows easily from (2.1) that

2.20 $$\begin{aligned} \rho _n/\beta _n =O(1), \end{aligned}$$

and also that

2.21 $$\begin{aligned} \sum _{k=0}^\infty kn_k = O\left( \sum _{k=0}^{\infty }(k-1)kn_{\mathrm{{S}},k}\right) = O(n_{\mathrm {S}}). \end{aligned}$$

Hence, (2.3) implies that $\alpha _n\rightarrow 0$.

To be precise, (2.3) and (2.1) yield by (2.14) and ${\mathcal R}_0\rightarrow 1$,

2.22 $$\begin{aligned} \alpha _n = \frac{{\mathcal R}_0-1}{{\mathcal R}_0} \frac{\sum _{k=0}^{\infty }(k-1) k n_{\mathrm {S},k}}{n_{\mathrm {S}}} =\bigl (1+o(1)\bigr )\lambda _2({\mathcal R}_0-1). \end{aligned}$$

Note that by combining the two parts of the argument, we have shown that our assumptions (D1), (D2) and (D4) imply (2.20) and the complementary bounds (2.19) and (2.21). (This can also easily be seen using (2.2).)

### Remark 2.10

We saw in Remark 2.9 that (D1), (D2) and (D4) imply (2.21). Since $n-n_0\leqslant \sum _{k=0}^\infty kn_k$, it follows that $n-n_0=O(n_{\mathrm {S}})$. Hence, assumption (D7) is needed only to the exclude the rather trivial case that almost all of the population consist of isolated infective vertices, which cannot spread the epidemic. Note also that (D7) implies that it does not matter whether we use $n_{\mathrm {S}}$ or *n* in estimates such as (2.11).

### *G*(*n*, *p*) and *G*(*n*, *m*)

The results above apply to the graphs $G(n,p)$ and $G(n,m)$ by conditioning on the sequence of vertex degrees (which are now random), since given the vertex degrees, both $G(n,p)$ and $G(n,m)$ are uniformly distributed over all (simple) graphs with these vertex degrees. Moreover, if ${n\rightarrow \infty }$ and $p\sim \lambda /n$, or $m\sim n\lambda /2$, for some $\lambda >0$, then the degree distribution is asymptotically Poisson ${\text {Po}}(\lambda )$. For $G(n,p)$, this leads to the following result.

#### Corollary 2.11

Suppose that $\beta _n > 0$ and $\rho _n \geqslant 0$ for each $n \in {\mathbb N}$. Let $\lambda \geqslant 1$, and assume that $\frac{\beta _n + \rho _n}{\beta _n} \rightarrow \lambda$ as $n \rightarrow \infty$. Let $\eta _n \rightarrow 0$, and consider the SIR epidemic on the random graph $G(n, \frac{\lambda (1+ \eta _n)}{n})$ with infection rate $\beta _n$ and recovery rate $\rho _n$. Suppose that there are $n_{\mathrm {I}}= o(n)$ initially infective vertices chosen at random, and all the other vertices are susceptible. Let

2.23 $$\begin{aligned} \gamma _n := 1 -\frac{\beta _n + \rho _n}{\lambda \beta _n}+\eta _n - (1+\eta _n)\frac{n_{\mathrm {I}}}{n}. \end{aligned}$$

Then $\gamma _n \rightarrow 0$. Assume that $n \gamma _n^3 \rightarrow \infty$, and that $\mu = \lim \frac{n_{\mathrm {I}}}{n \gamma ^2_n}$ exists.

(i) If $\mu =0$, then there exists a sequence $\varepsilon _n\rightarrow 0$ such that for each *n*, w.h.p. one of the following holds.
  - $${\mathcal Z}/(n \gamma _n) < \varepsilon _n $$.
  - $$|{\mathcal Z}/(n \gamma _n) -2| < \varepsilon _n$$.
  Moreover, the probability that (a) holds is
  2.24 $$\begin{aligned} \exp \bigl (-(1+\lambda ^{-1})\gamma _n n_I\bigr ) +o(1). \end{aligned}$$
  In particular, (a) holds w.h.p. if $\gamma _nn_{\mathrm {I}}\rightarrow 0$ and (b) holds w.h.p. if $\gamma _nn_{\mathrm {I}}\rightarrow \infty$.
(ii) If $0<\mu <\infty$, then ${\mathcal Z}/n \gamma _n \overset{\mathrm {p}}{\longrightarrow }1 + \sqrt{1 + 2\mu }$.
(iii) If $\mu = \infty$, then
  $$\begin{aligned} \frac{{\mathcal Z}}{(n_{\mathrm {S}}n_{\mathrm {I}})^{1/2}} \overset{\mathrm {p}}{\longrightarrow }\sqrt{2}. \end{aligned}$$

The same holds for $G(n,m)$ with $m=n\lambda (1+\eta _n)/2$.

#### Proof

As said above, we condition on the vertex degrees. We have $n_{\mathrm {S},k}/n_{\mathrm {S}}\overset{\mathrm {p}}{\longrightarrow }p_k:=\mathbb {P}{}({\text {Po}}(\lambda )=k)$ for every *k*; for convenience, we use the Skorohod coupling theorem (Kallenberg 2002, Theorem 4.30) so we may assume that this holds a.s. for each *k*; thus (2.4) holds a.s. Similarly we may assume that $\sum _k k^4n_{k}/n$ converges a.s., and then (D2) and (D3) hold a.s. Furthermore, $\alpha _n$ is now random, and it is easy to see from (2.2) that

2.25 $$\begin{aligned} \frac{n_{\mathrm {S}}}{n}\alpha _n= & {} -\frac{\beta _n+\rho _n}{\beta _n}(1+\eta _n)\lambda +\bigl ((1+\eta _n)\lambda \bigr )^2\Bigl (1-\frac{n_{\mathrm {I}}}{n}\Bigr )+O_p\bigl (n^{-1/2}\bigr ) \nonumber \\= & {} (1+\eta _n)\lambda ^2 \gamma _n+O_p\bigl (n^{-1/2}\bigr ) =\bigl (\lambda ^2+o_{\mathrm p}(1)\bigr )\gamma _n. \end{aligned}$$

Repeating the Skorohod trick, we may thus assume also that $\alpha _n/\gamma _n\rightarrow \lambda ^2$. Similarly we may assume $X_{\mathrm {I},0}=\sum _k kn_{\mathrm {I},k}=\bigl (1+O(n_{\mathrm {I}}^{-1/2})\bigr )\lambda {n_{\mathrm {I}}}$, and then (2.8) holds with $\nu =\mu /\lambda ^3$; it is also easy to see that (2.9) may be assumed. Then all the conditions (D1)–(D7) hold a.s., and the result follows as a consequence of Theorems 2.4 and 2.5, noting that $D_{\mathrm {S}}\sim {\text {Po}}(\lambda )$, and thus $\lambda _2=\lambda ^2$ and $\lambda _3=\lambda ^3$. $\square$

## Proof of Theorem 2.4

### Simplifying assumptions

We assume for convenience that $n_{\mathrm {I}}= o(n)$. In fact, we may assume that $n_{\mathrm {I},0}=0$ by deleting all initially infective vertices of degree 0, since these are irrelevant; then $n_{\mathrm {I}}=o(n)$ as a consequence of (2.7). Note that this will not affect ${\mathcal R}_0$, $\alpha _n$, $\nu$ or the other constants and assumptions above.

Similarly, we assume that initially there are no recovered vertices, that is $n_{\mathrm {R}}= 0$. It is easy to modify the proofs below to handle the case $n_{\mathrm {R}}\geqslant 1$. Alternatively, we may observe that our results in the case $n_{\mathrm {R}}= 0$ imply the corresponding results for general $n_{\mathrm {R}}$ by the following argument. (See Janson 2009a for similar arguments in a related situation.) We replace each initially recovered vertex of degree *k* by *k* separate susceptible vertices of degree 1, so there are a total of $X_{\mathrm {R},0}:=\sum _{k=0}^\infty kn_{\mathrm {R},k}$ additional “fake” susceptible vertices of degree 1; this will not change the course of the epidemic (in the multigraph case) except that some of these fake susceptible vertices will be infected. (Note that they never can infect anyone else.) The alteration will not affect ${\mathcal R}_0$, although $\alpha _n$ and the asymptotic distribution $(p_k)$ will be modified. Note that $X_{\mathrm {R},0}=O(n_{\mathrm {S}})$ by (D3) and (D7); by considering suitable subsequences we may thus assume that $X_{\mathrm {R},0}/n_{\mathrm {S}}\rightarrow r$ for some $r\in [0,\infty )$. It is easy see that the modified degree distribution satisfies all the assumptions above and that, if we use a prime to indicate quantities after the replacement, then $n_{\mathrm {S}}'=n_{\mathrm {S}}+X_{\mathrm {R},0}\sim (1+r)n_{\mathrm {S}}$, $\alpha _n'\sim \alpha _n/(1+r)$, $n_{\mathrm {S}}'\alpha _n'=n_{\mathrm {S}}\alpha _n$, $\nu '=(1+r)\nu$, $p_1'=(p_1+r)/(1+r)$, $p_k'=p_k/(1+r)$ for $k\ne 1$, $\lambda '=(\lambda +r)/(1+r)$, $\lambda _2'=\lambda _2/(1+r)$, $\lambda _3'=\lambda _3/(1+r)$.

If case (i)(a) in Theorem 2.4 occurs for the modified process, it occurs for the original process too, since ${\mathcal Z}\leqslant {\mathcal Z}'$, and there is nothing more to prove.

In the other cases, we have ${\mathcal Z}'\rightarrow \infty$ w.h.p. We note that of the $n_{\mathrm {S},1}'=n_{\mathrm {S},1}+X_{\mathrm {R},0}$ susceptible vertices of degree 1, $X_{\mathrm {R},0}$ are fake. Conditioned on the number ${\mathcal Z}_1'$ of susceptible vertices of degree 1 that get infected, the number ${\mathcal Z}'-{\mathcal Z}={\mathcal Z}_1'-{\mathcal Z}_1$ of fake susceptible vertices that get infected has a hypergeometric distribution, and, using e.g. Chebyshev’s inequality, it follows that w.h.p. (leaving the simple modification when $p_1=r=0$ to the reader)

3.1 $$\begin{aligned} {\mathcal Z}'-{\mathcal Z}={\mathcal Z}_1'-{\mathcal Z}_1=\frac{X_{\mathrm {R},0}}{n_{\mathrm {S},1}+X_{\mathrm {R},0}}{\mathcal Z}_1'+o({\mathcal Z}') =\frac{r}{p_1+r}{\mathcal Z}_1'+o({\mathcal Z}'). \end{aligned}$$

By (2.17) and the relations above, this yields w.h.p.

3.2 $$\begin{aligned} {\mathcal Z}'-{\mathcal Z}=\frac{r}{p_1+r}{\mathcal Z}_1'+o({\mathcal Z}') =\frac{r}{p_1+r}\frac{p_1'}{\lambda '}{\mathcal Z}'+o({\mathcal Z}') =\frac{r}{\lambda +r}{\mathcal Z}'+o({\mathcal Z}'). \end{aligned}$$

Consequently, w.h.p. $${\mathcal Z}/{\mathcal Z}'=\lambda /(\lambda +r)+o(1)$$.

It is then easy to check that Theorem 2.4 and Theorem 2.5 for the original process both follow from these results in the case with no initially recovered vertices.

We make these simplifying assumptions $n_{\mathrm {I}}=o(n)$ and $n_{\mathrm {R}}=0$ throughout this section (and the following one), in addition to (D1)–(D7). In particular, $n_{\mathrm {I}}+n_{\mathrm {R}}=o(n)$, and thus (D7) is strengthened to

3.3 $$\begin{aligned} n_{\mathrm {S}}/n \rightarrow 1. \end{aligned}$$

We may also assume $\alpha _n>0$, by ignoring some small *n* if necessary. Finally, recall that in the proofs we first consider the random multigraph $G^*$.

### Time-changed epidemic on a random multigraph

We first study the epidemic on the configuration model multigraph $G^*$, revealing its edges (i.e. pairing off the half-edges) while the epidemic spreads, as in Janson et al. (2014) (see other variants in Andersson 1998; Ball and Neal 2008; Decreusefond et al. 2012; Bohman and Picollelli 2012). We call a half-edge susceptible, infective or recovered according to the type of vertex it is attached to. Unpaired half-edges are said to be *free*. Initially, each vertex *i* has $d_i$ half-edges and all of them are free.

Each free infective half-edge chooses a free half-edge at rate $\beta _n > 0$, uniformly at random from among all the other free half-edges. Together the pair form an edge, and are removed from the set of free half-edges. If the chosen free half-edge belongs to a susceptible vertex then that vertex becomes infective. Infective vertices recover at rate $\rho _n \geqslant 0$.

We stop the process when no infective free half-edges remain, which is the time when the epidemic stops spreading. Some infective vertices may remain but they trivially recover at i.i.d. exponential times. Some free susceptible and recovered half-edges may also remain. These could be paired uniformly to reveal the remaining edges in $G^*$, if desired. However, this step is irrelevant for the course of the epidemic.

In order to prove our results, we perform a time change in the process: when in a state with $x_{\mathrm {I}}\geqslant 1$ free infective half-edges, and a total of *x* free half-edges of any type, we multiply all transition rates by $(x-1)/\beta _n x_{\mathrm {I}}$ (this multiple is at least $1/(2\beta _n)$, since $x_{\mathrm {I}}\geqslant 1$ implies that $x \geqslant 2$). Then each free susceptible half-edge gets infected at rate 1, each infective vertex recovers at rate $\rho _n(x-1)/\beta _nx_{\mathrm {I}}$, and each free infective half-edge pairs off at rate $(x-1)/x_{\mathrm {I}}$.

In the time changed process, let $S_{t}$, $I_{t}$ and $R_{t}$ denote the numbers of susceptible, infective and recovered vertices, respectively, at time $t \geqslant 0$. Let $S_{t}(k)$ be the number of susceptible vertices of degree $k\geqslant 0$ at time *t*. Then $S_{t} = \sum _{k=0}^\infty S_{t}(k)$ is decreasing and $R_{t}$ is increasing in *t*. Moreover, $S_{0}(k) = n_{\mathrm {S},k}$, $I_{0} = n_{\mathrm {I}}$ and $R_{0} = n_{\mathrm {R}}= 0$. Also, we let $X_{\mathrm {S},t}$, $X_{\mathrm {I},t}$ and $X_{\mathrm {R},t}$ be the numbers of free susceptible, infective and recovered half-edges, respectively, at time *t*. Then $X_{\mathrm {S},t} = \sum _{k=0}^\infty k S_{t}(k)$ is decreasing, $X_{\mathrm {S},0} = \sum _{k=0}^\infty k n_{\mathrm {S},k}$, $X_{\mathrm {I},0} = \sum _{k=0}^\infty k n_{\mathrm {I},k}$ and $X_{\mathrm {R},0} = 0$ (by our simplifying assumptions in Sect. 3.1).

We denote the duration of the time-changed epidemic by

3.4 $$\begin{aligned} T^*{:=} \inf \{t \geqslant 0 : X_{\mathrm {I},t} = 0 \}. \end{aligned}$$

At time $T^*$, we simply stop, as said above. (The last infection may have occurred somewhat earlier, since the last free infective half-edge may have recovered or paired of with an infective or recovered half-edge. It follows e.g. from (3.10) below that the last actual infection w.h.p. did not happen much earlier, but this is irrelevant for our results, and we use (3.4) as the definition.)

### Concentration of measure

We will show that $S_{t}(k)$, $X_{\mathrm {S},t}$, $X_{\mathrm {I},t}$ and $X_{\mathrm {R},t}$ are uniformly close to certain deterministic functions. Let

3.5 $$\begin{aligned} h_{\mathrm {S},n}(t)&:= \sum _{k=0}^\infty k n_{\mathrm {S},k}e^{-kt}, \end{aligned}$$

3.6 $$\begin{aligned} h_{\mathrm {R},n}(t)&:= \frac{\rho _n}{\beta _n}e^{-t}(1 - e^{-t})\sum _{k=0}^\infty kn_{k}, \end{aligned}$$

3.7 $$\begin{aligned} h_{\mathrm {I},n}(t)&:= e^{-2t}\sum _{k=0}^\infty k n_{k}- h_{\mathrm {S},n}(t) - h_{\mathrm {R},n}(t). \end{aligned}$$

#### Theorem 3.1

Let $\tilde{\alpha }_n$ be any numbers with $\alpha _n\leqslant \tilde{\alpha }_n=o(1)$ such that

3.8 $$\begin{aligned} \sum _{k=0}^\infty k^2 n_{\mathrm {I},k}= o\bigl (n^2\tilde{\alpha }_n^4\bigr ). \end{aligned}$$

Then, for any fixed $t_0 <\infty$,

3.9 $$\begin{aligned}&\sum _{k=0}^\infty \sup _{t\leqslant \tilde{\alpha }_nt_0 \wedge T^*} |S_{t}(k) - n_{\mathrm {S},k}e^{-kt}| = o_{\mathrm p}(n\tilde{\alpha }_n^2), \end{aligned}$$

3.10 $$\begin{aligned}&\sup _{t\leqslant \tilde{\alpha }_nt_0 \wedge T^*} |X_{\mathrm {S},t} - h_{\mathrm {S},n}(t)| = o_{\mathrm p}(n\tilde{\alpha }_n^2), \end{aligned}$$

3.11 $$\begin{aligned}&\sup _{t\leqslant \tilde{\alpha }_nt_0 \wedge T^*} |X_{\mathrm {R},t} - h_{\mathrm {R},n}(t)| = o_{\mathrm p}(n\tilde{\alpha }_n^2), \end{aligned}$$

3.12 $$\begin{aligned}&\sup _{t\leqslant \tilde{\alpha }_nt_0 \wedge T^*} |X_{\mathrm {I},t} - h_{\mathrm {I},n}(t)| = o_{\mathrm p}(n\tilde{\alpha }_n^2). \end{aligned}$$

The above result establishes concentration on time intervals of length $O(\tilde{\alpha }_n)$. In Sect. 3.4, we use it to show that, for a suitable choice of $\tilde{\alpha }_n$, the duration of the epidemic satisfies $T^*=O(\tilde{\alpha }_n)$ w.h.p. It follows that the theorem then holds also with $t_0=\infty$, see Remark 3.6.

The remainder of this subsection contains the proof of Theorem 3.1. We first need two lemmas concerning the evolution of the number of susceptible vertices and the total number of free half-edges.

In the time-changed epidemic, each free susceptible half-edge gets infected at rate 1, until $T^*$. We further modify the process so that free susceptible half-edges continue to be infected at rate 1 even when there are no more free infective half-edges. Let $\tilde{S}_{t}(k)$ be the number of susceptible individuals of degree *k* in the modified process. Then $(\tilde{S}_{t\wedge T^*}(k): k \in {\mathbb Z}^+, t \geqslant 0)$ has the same distribution as $(S_{t\wedge T^*}(k): k \in {\mathbb Z}^+, t \geqslant 0)$, and so, to prove (3.9) and (3.10), it suffices to prove that

3.13 $$\begin{aligned} \sum _{k=0}^\infty \sup _{t\leqslant \tilde{\alpha }_nt_0} |\tilde{S}_{t}(k) - n_{\mathrm {S},k}e^{-kt}| = o_{\mathrm p}(n\tilde{\alpha }_n^2), \end{aligned}$$

and

3.14 $$\begin{aligned} \sup _{t\leqslant \tilde{\alpha }_nt_0} |\tilde{X}_{S,t} - h_{\mathrm {S},n}(t)| = o_{\mathrm p}(n\tilde{\alpha }_n^2), \end{aligned}$$

where $\tilde{X}_{S,t}= \sum _{k=0}^\infty k\tilde{S}_{t}(k)$. For each *t*, let

3.15 $$\begin{aligned} W_t := \sum _{k=0}^\infty k^2( \tilde{S}_{t}(k) - n_{\mathrm {S},k}e^{-kt} ). \end{aligned}$$

#### Lemma 3.2

Fix $t_0 <\infty$ and assume $\alpha _n\leqslant \tilde{\alpha }_n=o(1)$. Then $\mathbb {E}{}\sup _{t\leqslant \tilde{\alpha }_nt_0} |W_t|=o(n \tilde{\alpha }_n)$, and hence

$$\begin{aligned} \mathbb {E}{}\sup _{t\leqslant \tilde{\alpha }_nt_0\wedge T^*} \left| {\sum _{k=0}^\infty k^2( S_{t}(k) - n_{\mathrm {S},k}e^{-kt} )}\right| = o(n\tilde{\alpha }_n). \end{aligned}$$

#### Proof of Lemma 3.2

We enumerate the initially susceptible vertices as $i \!=\! 1,2,\ldots ,n_{\mathrm {S}}$ and denote by $d_{\mathrm {S},i}$ the degree of initially susceptible vertex *i*. Let $L_i$ be the time at which initially susceptible vertex *i* becomes infective (in the modified process). Then each $L_i$ has exponential distribution with rate $d_{\mathrm {S},i}$, and the $L_i$ ($i = 1,2,\ldots ,n_{\mathrm {S}}$) are all independent of one another. It follows that, for each fixed *t*, the random variables $F_{i,t}{:=} d_{\mathrm {S},i}^2(\mathop {\mathbbm {1}_{L_i > t}}\nolimits - e^{-td_{\mathrm {S},i}})$ each have mean zero and are all independent. Note that $W_t = \sum _{i = 1}^{n_{\mathrm {S}}} F_{i,t}$.

Each $|F_{i,t}|$ is bounded by $d_{\mathrm {S},*}^2$, where, as in (2.11), $d_{\mathrm {S},*}= \max _id_{\mathrm {S},i}$. Hence, by Bernstein’s inequality for sums of bounded independent centred random variables, see e.g. McDiarmid (1998, Theorem 2.7)] or Boucheron et al. (2013, 2.10), for each $a\geqslant 0$,

3.16 $$\begin{aligned} \mathbb {P}(|W_t|> a) = \mathbb {P}\left( \left| {\sum _{i = 1}^{n_{\mathrm {S}}} F_{i,t}}\right| > a\right) \leqslant 2\exp \left( - \frac{a^2}{2\sum _{i = 1}^{n_{\mathrm {S}}} \mathbb {E}{}F_{i,t}^2 + 2a d_{\mathrm {S},*}^2/3} \right) . \end{aligned}$$

Now, for any $t \leqslant \tilde{\alpha }_nt_0$, using (2.10),

3.17 $$\begin{aligned} \begin{aligned} 2\sum _{i = 1}^{n_{\mathrm {S}}} \mathbb {E}{}F_{i,t}^2&= 2\sum _{i = 1}^{n_{\mathrm {S}}} d_{\mathrm {S},i}^4{\text {Var}}\bigl (\mathop {\mathbbm {1}_{L_i > t}}\nolimits \bigr ) \leqslant 2\sum _{i = 1}^{n_{\mathrm {S}}} d_{\mathrm {S},i}^4 \bigl (1 - e^{-td_{\mathrm {S},i}}\bigr )\\&\leqslant 2 d_{\mathrm {S},*}^2 t \sum _{i = 1}^{n_{\mathrm {S}}} d_{\mathrm {S},i}^3 \leqslant 2t_0 \tilde{\alpha }_nd_{\mathrm {S},*}^2 \sum _k k^3n_{\mathrm {S},k}\leqslant 2c_0t_0 \tilde{\alpha }_nn d_{\mathrm {S},*}^2. \end{aligned} \end{aligned}$$

Furthermore, $\tilde{\alpha }_nn^{1/3}\geqslant \alpha _n n^{1/3}\rightarrow \infty$ and so by (2.11),

3.18 $$\begin{aligned} d_{\mathrm {S},*}= o\bigl (n^{1/3}\bigr ) =o\bigl ((n\tilde{\alpha }_n)^{1/2}\bigr ). \end{aligned}$$

Thus, for *n* sufficiently large, $d_{\mathrm {S},*}\leqslant (n\tilde{\alpha }_n)^{1/2}$, and then for any $u \geqslant 2c_0 t_0$ and $a = u (n \tilde{\alpha }_n)^{1/2}d_{\mathrm {S},*}$, by (3.17),

$$\begin{aligned} \begin{aligned} \exp \left( - \frac{a^2}{2\sum _{i = 1}^{n_{\mathrm {S}}} \mathbb {E}{}F_{i,t}^2 + 2a d_{\mathrm {S},*}^2/3} \right)&\leqslant \exp \left( - \frac{u^2}{2c_0t_0 + 2u d_{\mathrm {S},*}/3(n\tilde{\alpha }_n)^{1/2}} \right) \\&\leqslant \exp \left( - \frac{u^2}{2c_0t_0 + u} \right) \leqslant \exp \left( - u/2\right) . \end{aligned} \end{aligned}$$

Hence, by (3.16), for *n* sufficiently large and for each each $t \leqslant t_0 \tilde{\alpha }_n$ and $u \geqslant 2c_0t_0$,

3.19 $$\begin{aligned} \mathbb {P}(|W_t| > u (n \tilde{\alpha }_n)^{1/2}d_{\mathrm {S},*}) \leqslant 2\exp \left( - u/2\right) . \end{aligned}$$

Note also that $(n \tilde{\alpha }_n)^{1/2} d_{\mathrm {S},*}= o(n \tilde{\alpha }_n)$ by (3.18). Let $\omega _n$ be an integer valued function such that $\omega _n \rightarrow \infty$ and $(n \tilde{\alpha }_n)^{1/2} d_{\mathrm {S},*}\omega _n = o(n \tilde{\alpha }_n)$. We divide the interval $[0,t_0 \tilde{\alpha }_n]$ into $\omega _n$ subintervals $[\tau _l,\tau _{l+1}]$, where $\tau _l = l t_0\tilde{\alpha }_n/\omega _n$ for $l=0,\ldots , \omega _n-1$.

Since $\tilde{S}_{t}(k)$ and $e^{-kt}$ are both decreasing in *t*, each of the sums $\sum _{k=0}^\infty k^2 \tilde{S}_{t}(k)$ and $\sum _{k=0}^\infty k^2 n_{\mathrm {S},k}e^{-kt}$ is also decreasing in *t*. Thus, for any $0 \leqslant l < \omega _n$,

$$\begin{aligned} \sup _{\tau _l \leqslant t \leqslant \tau _{l+1}}|W_t|&\leqslant \left| \sum _{k=0}^\infty k^2(\tilde{S}_{\tau _l}(k) - n_{\mathrm {S},k}e^{-k\tau _{l+1}})\right| + \left| \sum _{k=0}^\infty k^2(\tilde{S}_{\tau _{l+1}}(k) - n_{\mathrm {S},k}e^{-k\tau _{l}})\right| \\&\leqslant |W_{\tau _{l}}| + |W_{\tau _{l +1}}| + 2\sum _{k=0}^\infty k^2 n_{\mathrm {S},k}(e^{-k\tau _l} - e^{-k\tau _{l+1}})\\&\leqslant |W_{\tau _{l}}| + |W_{\tau _{l +1}}| + 2\sum _{k=0}^\infty k^3 n_{\mathrm {S},k}(\tau _{l+1} - \tau _l ),\end{aligned}$$

and so, since $\sum _{k=0}^\infty k^3 n_{\mathrm {S},k}\leqslant c_0 n$ and $\tau _{l+1}-\tau _l=t_0\tilde{\alpha }_n/\omega _n=o(\tilde{\alpha }_n)$, noting $W_0=0$,

3.20 $$\begin{aligned} \sup _{t \leqslant \tilde{\alpha }_nt_0}|W_t| = \max _{l < \omega _n} \sup _{\tau _l \leqslant t \leqslant \tau _{l+1}}|W_t| \leqslant 2\max _{1 \leqslant l \leqslant \omega _n}|W_{\tau _{l}}| + o(n\tilde{\alpha }_n). \end{aligned}$$

Now, for *n* sufficiently large and $u \geqslant 2c_0t_0$, by (3.19),

3.21 $$\begin{aligned} \mathbb {P}\left( \max _{1 \leqslant l\leqslant \omega _n}|W_{\tau _{l}}| > u(n \tilde{\alpha }_n)^{1/2}d_{\mathrm {S},*}\right) \leqslant 2\omega _n \exp (-u/2). \end{aligned}$$

For sufficiently large *n*, $2\omega _n\geqslant e^{c_0t_0}$, and then (3.21) holds trivially for $u<2c_0t_0$ too. Hence, for large *n*,

3.22 $$\begin{aligned} \mathbb {E}{}\max _{1 \leqslant l \leqslant \omega _n } | W_{\tau _{l}} |&= (n\tilde{\alpha }_n)^{1/2}d_{\mathrm {S},*}\int _{0}^\infty \mathbb {P}\left( \max _{1 \leqslant l \leqslant \omega _n} |W_{\tau _{l}}| > u (n \tilde{\alpha }_n)^{1/2}d_{\mathrm {S},*}\right) \,du \nonumber \\&\leqslant (n \tilde{\alpha }_n)^{1/2}d_{\mathrm {S},*}\int _{0}^\infty 2\omega _n e^{-u/2} du \nonumber \\&=4 (n \tilde{\alpha }_n)^{1/2}d_{\mathrm {S},*}\omega _n = o(n\tilde{\alpha }_n) , \end{aligned}$$

and hence also $\mathbb {E}{}\sup _{t \leqslant \tilde{\alpha }_nt_0}|W_t| = o(n \tilde{\alpha }_n)$ by (3.20). $\square$

We now prove a concentration of measure result for the total number $X_{t}$ of free half-edges.

#### Lemma 3.3

For every fixed $t_0 > 0$, and $\alpha _n\leqslant \tilde{\alpha }_n=o(1)$,

3.23 $$\begin{aligned} \sup _{t \leqslant \tilde{\alpha }_nt_0 \wedge T^*} \left| {X_{t} - e^{-2t}\sum _{k=0}^\infty kn_{k}}\right| = o_{\mathrm p}(n\tilde{\alpha }_n^2). \end{aligned}$$

#### Proof

When in a state with $x_{\mathrm {I}}\geqslant 1$ free infective half-edges, and thus $x \geqslant 2$ free half-edges in total, each free infective half-edge pairs off at rate $(x-1)/x_{\mathrm {I}}$, and so the number of free half-edges decreases by 2 at rate $x-1$. We modify the process so that pairs of free half-edges still disappear at rate $x-1$ when there are no more free infective half-edges (as long as $x\geqslant 2$). Let $\tilde{X}_t$ be the number of free half-edges at time *t* in the modified process. Then it suffices to prove that

$$\begin{aligned} \sup _{t \leqslant \tilde{\alpha }_nt_0} \left| {\tilde{X}_t - e^{-2t}\sum _{k=0}^\infty kn_{k}}\right| = o_{\mathrm p}(n\tilde{\alpha }_n^2). \end{aligned}$$

Now, $\tilde{X_{t}}-1$ is a linear death chain starting from $\sum _{k=0}^\infty kn_{k}- 1$, and taking jumps from state *j* to $j -2$ at rate *j*. By Janson and Luczak (2009, Lemma 6.1), with $d = 2$, $\gamma = 1$, and $x = \sum _{k=0}^\infty kn_{k}- 1$,

$$\begin{aligned} \mathbb {E}{}\sup _{t \leqslant \tilde{\alpha }_nt_0} \left| { (\tilde{X_{t}}-1) - e^{-2t}\left( \sum _{k=0}^{\infty }kn{_{k}}{-1}\right) }\right| ^2 \leqslant 16 (e^{2\tilde{\alpha }_nt_0} - 1)\sum _{k=0}^\infty kn_{k}+ 32. \end{aligned}$$

But $\sum _{k=0}^\infty kn_{k}= O(n)$ by (D3), $\tilde{\alpha }_nt_0 = o(1)$ and $n\tilde{\alpha }_n\geqslant n\alpha _n\rightarrow \infty$ by (D4), so the right-hand side is $O(n\tilde{\alpha }_n)$, and so $\sup _{t \leqslant \tilde{\alpha }_nt_0} \bigl |\tilde{X}_t - e^{-2t}\sum _{k=0}^\infty kn_{k}\bigr | = O_{\mathrm p}\bigl (\sqrt{n \tilde{\alpha }_n}\bigr ) = o_{\mathrm p}(n\tilde{\alpha }_n^2)$, using (D4). $\square$

#### Proof of Theorem 3.1

We start by proving (3.10), and, as remarked after the statement of Theorem 3.1, it is enough to prove that

3.24 $$\begin{aligned} \sup _{t\leqslant \tilde{\alpha }_nt_0 } \left| {\sum _{k=0}^\infty k\tilde{S}_{t}(k) - h_{\mathrm {S},n}(t)}\right| = o_{\mathrm p}(n\tilde{\alpha }_n^2). \end{aligned}$$

Now, for each *k*, $(\tilde{S}_{t}(k))$ is a linear death chain starting from $n_{\mathrm {S},k}$ and decreasing by 1 at rate *kx* when in state *x*, and so

3.25 $$\begin{aligned} \tilde{S}_{t}(k) = n_{\mathrm {S},k}- k \int _0^t \tilde{S}_{u}(k)du + \tilde{M}_{t}(k), \end{aligned}$$

where $\tilde{M}(k)=(\tilde{M}_t(k))$ is a zero-mean martingale. It follows that

3.26 $$\begin{aligned} \sum _{k=0}^\infty k \tilde{S}_{t}(k) - h_{\mathrm {S},n}(t)&= \sum _{k=0}^\infty k (\tilde{S}_{t}(k) - n_{\mathrm {S},k}e^{-kt}) \nonumber \\&= - \sum _{k=0}^\infty k^2 \int _0^t (\tilde{S}_{u}(k) - n_{\mathrm {S},k}e^{-ku})du + \tilde{M}_t \nonumber \\&= -\int _0^t W_u du + \tilde{M}_t, \end{aligned}$$

where $\tilde{M}_t = \sum _{k} k \tilde{M}_{t}(k)$ defines a zero-mean martingale $\tilde{M}= (\tilde{M}_t)$.

Since $\tilde{S}_{t}(k)$ and $\tilde{S}_t(j)$ with $k \not = j$ never jump simultaneously, we have $[\tilde{M}(k),\tilde{M}(j)]=0$, where $[\cdot ,\cdot ]$ is the quadratic covariation, see e.g. Kallenberg (2002, Theorem 26.6). Since also each jump of $\tilde{S}_{t}(k)$ is by $-1$, the quadratic variation $[\tilde{M}]_t:=[\tilde{M},\tilde{M}]_t$ is

$$\begin{aligned}{}[\tilde{M}]_t&= \sum _{k=0}^\infty k^2 [M(k)]_t = \sum _{k=0}^\infty k^2 \sum _{u \leqslant t} (\Delta \tilde{S}_{u}(k))^2 \\&= - \sum _{k=0}^\infty k^2 \sum _{u \leqslant t} (\Delta \tilde{S}_{u}(k)) = \sum _{k=0}^\infty k^2 (n_{\mathrm {S},k}- \tilde{S}_{t}(k)) \\&= \sum _{k=0}^\infty k^2 (n_{\mathrm {S},k}e^{-kt} - \tilde{S}_{t}(k) + n_{\mathrm {S},k}(1 - e^{-kt})) \\&\leqslant \left| {\sum _{k=0}^\infty k^2 (\tilde{S}_{t}(k) - n_{\mathrm {S},k}e^{-kt})}\right| + t\sum _{k=0}^\infty k^3 n_{\mathrm {S},k}\\&= |W_t| + O(tn), \end{aligned}$$

again using (2.10). By Lemma 3.2, $\mathbb {E}{}\sup _{t \leqslant \tilde{\alpha }_nt_0}|W_t| = o(n\tilde{\alpha }_n)$, so

3.27 $$\begin{aligned} \mathbb {E}{}[\tilde{M}]_{\tilde{\alpha }_nt_0} = O(n \tilde{\alpha }_n), \end{aligned}$$

and so, using the Burkholder–Davis–Gundy inequalities (Kallenberg 2002, Theorem 26.12) $\sup _{t \leqslant \tilde{\alpha }_nt_0} |\tilde{M}_t| = O_{\mathrm p}(\sqrt{n\tilde{\alpha }_n})$. Hence by (3.26) and Lemma 3.2, uniformly in $t \leqslant \tilde{\alpha }_nt_0$,

$$\begin{aligned} \left| \sum _{k=0}^\infty k (\tilde{S}_{t}(k) - n_{\mathrm {S},k}e^{-kt})\right|&\leqslant \left| \int _0^t W_u du\right| + O_{\mathrm p}(\sqrt{n\tilde{\alpha }_n}) \\&\leqslant \tilde{\alpha }_nt_0 \sup _{t \leqslant \tilde{\alpha }_nt_0} |W_t| + O_{\mathrm p}(\sqrt{n\tilde{\alpha }_n}) \\&= o_{\mathrm p}(n \tilde{\alpha }_n^2), \end{aligned}$$

using again (D4). This establishes (3.10).

Next we prove (3.9). By (Janson and Luczak 2009, Lemma 6.1) with $d = 1$ and $\gamma = k$ and $x = n_{\mathrm {S},k}$, for $k \leqslant \tilde{\alpha }_n^{-1}$,

3.28 $$\begin{aligned} \mathbb {E}{}\sup _{t \leqslant \tilde{\alpha }_nt_0} |\tilde{S}_{t}(k) - n_{\mathrm {S},k}e^{-kt}|^2 \leqslant 4 ( e^{k\tilde{\alpha }_nt_0} - 1) n_{\mathrm {S},k}\leqslant 4 k\tilde{\alpha }_nt_0 e^{t_0} n_{\mathrm {S},k}, \end{aligned}$$

where the last step uses the simple inequality $e^x-1\leqslant xe^x$ for $x\geqslant 0$. For $k > \tilde{\alpha }_n^{-1}$, we use the trivial bound $|\tilde{S}_{t}(k) - n_{\mathrm {S},k}e^{-kt}| \leqslant n_{\mathrm {S},k}$. Using Jensen’s inequality and then the Cauchy–Schwarz inequality, as well as (2.10) and (D4),

$$\begin{aligned}&\mathbb {E}{}\sum _{k=0}^\infty \sup _{t \leqslant \tilde{\alpha }_nt_0} |\tilde{S}_{t}(k) - n_{\mathrm {S},k}e^{-kt}| \leqslant \sum _{1\leqslant k \leqslant \tilde{\alpha }_n^{-1}} (4 k\tilde{\alpha }_nt_0 e^{t_0} n_{\mathrm {S},k})^{1/2} + \sum _{k > \tilde{\alpha }_n^{-1}} n_{\mathrm {S},k}\\&\quad \leqslant 2(\tilde{\alpha }_nt_0 e^{t_0})^{1/2} \left( \sum _{k=1}^\infty k^{-2} \sum _{k=1}^\infty k^{3} n_{\mathrm {S},k}\right) ^{1/2} + \tilde{\alpha }_n^3 \sum _{k=1}^\infty k^3 n_{\mathrm {S},k}\\&\quad = O( \sqrt{n\tilde{\alpha }_n}) + O( n\tilde{\alpha }_n^3) = o(n\tilde{\alpha }_n^2), \end{aligned}$$

which yields (3.13) and thus (3.9).

We now prove (3.11). The number of free recovered half-edges changes when either an infective vertex recovers or a free infective half-edge pairs with a free recovered half-edge. In the time-changed process, when in a state with $x_{\mathrm {I}}$ free infective half-edges and *x* free half-edges, infective vertices recover at rate $\rho _n (x-1)/\beta _n x_{\mathrm {I}}$. Also, each free recovered half-edge is chosen to be paired at rate 1, and thus the number of recovered free half-edges decreases by 1 at rate $x_{\mathrm {R}}$. Hence, for any $t\geqslant 0$,

3.29 $$\begin{aligned} X_{\mathrm {R},t \wedge T^*} = X_{\mathrm {R},0} - \int _0^{t \wedge T^*} X_{\mathrm {R},s}ds + \frac{\rho _n}{\beta _n} \int _0^{t \wedge T^*}(X_{s} - 1) ds + M_{\mathrm {R}, t \wedge T^*}, \end{aligned}$$

where $M_{\mathrm {R}} = (M_{\mathrm {R},t})$ is a zero-mean martingale.

On the other hand, differentiating (3.6) reveals that

3.30 $$\begin{aligned} h_{\mathrm {R},n}'(t) = -h_{\mathrm {R},n}(t) + \frac{\rho _n}{\beta _n}e^{-2t} \sum _{k=0}^\infty kn_{k}. \end{aligned}$$

Hence, subtracting the integral of that expression from (3.29), and recalling that $X_{\mathrm {R},0}=0$,

$$\begin{aligned} \Bigl |X_{\mathrm {R},t\wedge T^*} -h_{\mathrm {R},n}(t\wedge T^*)\Bigr |&\leqslant \int _0^{t \wedge T^*}|X_{\mathrm {R},s\wedge T^*} -h_{\mathrm {R},n}(s\wedge T^*)|ds \\&\quad + \frac{\rho _n}{\beta _n}\int _0^{t \wedge T^*} \left| X_{s} - 1 - e^{-2s} \sum _{k=0}^\infty kn_{k}\right| ds + |M_{\mathrm {R}, t\wedge T^*}|. \end{aligned}$$

Then Gronwall’s inequality yields

3.31 $$\begin{aligned} \sup _{t \leqslant \tilde{\alpha }_nt_0\wedge T^*} |X_{\mathrm {R},t} - h_{\mathrm {R},n}(t)|\leqslant & {} e^{\tilde{\alpha }_nt_0} \tilde{\alpha }_nt_0 \frac{\rho _n}{\beta _n} \left( \sup _{t \leqslant \tilde{\alpha }_nt_0\wedge T^*} \Bigl |X_{t} - e^{-2t} \sum _{k=0}^\infty kn_{k}\Bigr | + 1\right) \nonumber \\&+ e^{\tilde{\alpha }_nt_0} \sup _{t \leqslant \tilde{\alpha }_nt_0\wedge T^*} |M_{\mathrm {R},t}|. \end{aligned}$$

Since $\rho _n/\beta _n$ is bounded and $\tilde{\alpha }_n\rightarrow 0$, the first term on the right-hand side is $o_{\mathrm p}(n\tilde{\alpha }_n^2)$, by (3.23). It remains to show that the same is true of the martingale term.

Note that $X_{R,t}$ jumps by $-1$ when a free recovered half-edge is paired with a free infective half-edge, and it jumps by $+k$ when an infective vertex with *k* free half-edges recovers. Also, each recovered half-edge or vertex was either initially infective or was initially susceptible and then became infected prior to recovery. Hence

$$\begin{aligned} \mathbb {E}{}[M_\mathrm {R}]_{\tilde{\alpha }_nt_0 \wedge T^*}&= \mathbb {E}{}\left[ \sum _{s \leqslant \tilde{\alpha }_nt_0 \wedge T^*} (\Delta M_{\mathrm {R},s})^2\right] \\&\leqslant \mathbb {E}{}\sum _{k=0}^\infty k \bigl (n_{\mathrm {I},k}+ n_{\mathrm {S},k}- S_{\tilde{\alpha }_nt_0 \wedge T^*}(k)\bigr )\\&\quad + \mathbb {E}{}\sum _{k=0}^\infty k^2 \bigl (n_{\mathrm {I},k}+ n_{\mathrm {S},k}- S_{\tilde{\alpha }_nt_0 \wedge T^*}(k)\bigr ) \\&\leqslant 2 \sum _{k=0}^\infty k^2 n_{\mathrm {I},k}+ 2\mathbb {E}{}\sum _{k=0}^\infty k^2\bigl (n_{\mathrm {S},k}- S_{\tilde{\alpha }_nt_0 \wedge T^*}(k)\bigr )\\&= 2 \sum _{k=0}^\infty k^2 n_{\mathrm {I},k}+ 2\mathbb {E}{}\sum _{k=0}^\infty k^2\bigl (n_{\mathrm {S},k}(1 - e^{-k(\tilde{\alpha }_nt_0 \wedge T^*)})\\&\quad +n_{\mathrm {S},k}e^{-k(\tilde{\alpha }_nt_0 \wedge T^*)} - S_{\tilde{\alpha }_nt_0 \wedge T^*}(k)\bigr )\\&\leqslant 2 \sum _{k=0}^\infty k^2 n_{\mathrm {I},k}+2\tilde{\alpha }_nt_0 \sum _{k=0}^\infty k^3 n_{\mathrm {S},k}\\&\quad + 2\mathbb {E}{}\sum _{k=0}^\infty k^2\bigl (n_{\mathrm {S},k}e^{-k(\tilde{\alpha }_nt_0 \wedge T^*)} - S_{\tilde{\alpha }_nt_0 \wedge T^*}(k)\bigr )\\&= o\bigl (n^2\tilde{\alpha }_n^4\bigr )+ O(n\tilde{\alpha }_n)+o(n\tilde{\alpha }_n) = o\bigl (n^2\tilde{\alpha }_n^4\bigr ) \end{aligned}$$

by (3.8), (2.10), Lemma 3.2 and $n\tilde{\alpha }_n^3\geqslant n\alpha _n^3\rightarrow \infty$, see (D4). Then, by the Burkholder–Davis–Gundy inequalities, $\sup _{t \leqslant \tilde{\alpha }_nt_0\wedge T^*} |M_{\mathrm {R},t}| = o_{\mathrm p}(n\tilde{\alpha }_n^2)$, and (3.11) follows by (3.31).

Finally, (3.12) follows from (3.10), (3.11), (3.23), and the fact that $X_{\mathrm {I},t} = X_{t} - X_{\mathrm {S},t} - X_{\mathrm {R},t}$. $\square$

### Duration of the time-changed epidemic

We stated Theorem 3.1 using a rather arbitrary $\tilde{\alpha }_n$, but from now on we fix it as follows. We distinguish between the cases $\nu <\infty$ and $\nu =\infty$, and introduce some further notation:

If $$0\leqslant \nu <\infty $$, define

3.32 $$\begin{aligned} \tilde{\alpha }_n&:=\alpha _n, \end{aligned}$$

3.33 $$\begin{aligned} f(t)&:=\nu +t-\tfrac{\lambda _3}{2}t^2, \end{aligned}$$

3.34 $$\begin{aligned} \varkappa&:=(1 + \sqrt{1+2\nu \lambda _3})/\lambda _3. \end{aligned}$$

If $\nu =\infty$, define instead

3.35 $$\begin{aligned} \tilde{\alpha }_n&:=\left( \sum _{k=0}^{\infty }{kn_{\mathrm{{I},}k}/n}\right) ^{1/2}, \end{aligned}$$

3.36 $$\begin{aligned} f(t)&:=1-\tfrac{\lambda _3}{2}t^2, \end{aligned}$$

3.37 $$\begin{aligned} \varkappa&:=\sqrt{2/\lambda _3}. \end{aligned}$$

Note that in both cases, $\varkappa$ is the unique positive root of *f*, and that $f(t)>0$ on $(0,\varkappa )$ and $f(t)<0$ on $(\varkappa ,\infty )$; we have $f(0)=0$ if $\nu =0$ but $f(0)>0$ if $\nu >0$. Note further that in the case $\nu =\infty$, $\tilde{\alpha }_n/\alpha _n\rightarrow \infty$ by (2.8); in particular, $\tilde{\alpha }_n\geqslant \alpha _n$ except possibly for some small *n* that we will ignore. Moreover, $\tilde{\alpha }_n\rightarrow 0$ by (D4) ($\nu <\infty$) or (D5) ($\nu =\infty$).

Next, if $\nu <\infty$, then, by (2.8),

3.38 $$\begin{aligned} \sum _{k=0}^\infty kn_{\mathrm {I},k}= O\bigl (n_{\mathrm {S}}\alpha _n^2\bigr )= O\bigl (n\tilde{\alpha }_n^2\bigr ), \end{aligned}$$

and if $\nu =\infty$, then by (3.35),

3.39 $$\begin{aligned} \sum _{k=0}^\infty kn_{\mathrm {I},k}= n\tilde{\alpha }_n^2. \end{aligned}$$

Hence, in both cases,

3.40 $$\begin{aligned} \sum _{k=0}^\infty kn_{\mathrm {I},k}= O\bigl (n\tilde{\alpha }_n^2\bigr ). \end{aligned}$$

Furthermore, if $\nu =0$ then (2.8) yields $\sum _k kn_{\mathrm {I},k}= o\bigl (n\alpha _n^2\bigr )$ and thus

3.41 $$\begin{aligned} \sum _{k=0}^\infty k^2n_{\mathrm {I},k}\leqslant \left( \sum _{k=0}^{\infty }kn_{\mathrm{{I}},k}\right) ^2 = o\bigl (n^2\tilde{\alpha }_n^4\bigr ), \end{aligned}$$

and if $0<\nu \leqslant \infty$ then (2.9) and (3.40) imply

3.42 $$\begin{aligned} \sum _{k=0}^\infty k^2n_{\mathrm {I},k}\leqslant d_{\mathrm {I},*}\sum _{k=0}^\infty kn_{\mathrm {I},k}=o \left( \sum _{k=0}^{\infty }kn_{\mathrm{{I}},k}\right) ^2 = o\bigl (n^2\tilde{\alpha }_n^4\bigr ). \end{aligned}$$

Hence, (3.8) holds in all cases.

We have verified that our choice of $\tilde{\alpha }_n$ satisfies the conditions of Theorem 3.1, so Theorem 3.1 applies. We use this to show a more explicit limit result for $X_{\mathrm {I},t}$.

#### Lemma 3.4

For any fixed $t_0$,

3.43 $$\begin{aligned} \sup _{t \leqslant t_0 \wedge (T^*/\tilde{\alpha }_n)} \left| \frac{X_{\mathrm {I},\tilde{\alpha }_nt}}{n\tilde{\alpha }_n^2} - f(t)\right| \overset{\mathrm {p}}{\longrightarrow }0. \end{aligned}$$

#### Proof

The idea is to combine Theorem 3.1 with a Taylor expansion of $h_{\mathrm {I},n}(t)$ around zero.

The first three derivatives of $h_{\mathrm {I},n}(t)$ are

3.44 $$\begin{aligned} h_{\mathrm {I},n}'(t)&= -2 e^{-2t} \sum _{k=0}^\infty kn_{k}+ \sum _{k=0}^\infty k^2 n_{\mathrm {S},k}e^{-kt} + \frac{\rho _n}{\beta _n}e^{-t}(1 - 2e^{-t})\sum _{k=0}^\infty kn_{k}, \end{aligned}$$

3.45 $$\begin{aligned} h_{\mathrm {I},n}''(t)&= 4 e^{-2t} \sum _{k=0}^\infty kn_{k}- \sum _{k=0}^\infty k^3 n_{\mathrm {S},k}e^{-kt} - \frac{\rho _n}{\beta _n}e^{-t}(1 - 4e^{-t})\sum _{k=0}^\infty kn_{k}, \end{aligned}$$

3.46 $$\begin{aligned} h_{\mathrm {I},n}'''(t)&= -8 e^{-2t} \sum _{k=0}^\infty kn_{k}+ \sum _{k=0}^\infty k^4 n_{\mathrm {S},k}e^{-kt} + \frac{\rho _n}{\beta _n}e^{-t}(1 - 8e^{-t})\sum _{k=0}^\infty kn_{k}. \end{aligned}$$

Hence, using (2.2), (3.3), (3.40) and $\tilde{\alpha }_n\rightarrow 0$,

3.47 $$\begin{aligned} h_{\mathrm {I},n}'(0) \!= \!-2 \sum _{k=0}^\infty kn_{k}\!+\! \sum _{k=0}^\infty k^2 n_{\mathrm {S},k}\!-\!\frac{\rho _n}{\beta _n}\sum _{k=0}^\infty kn_{k}\!= \!n_{\mathrm {S}}\alpha _n \!-\! \sum _{k=0}^\infty kn_{\mathrm {I},k}\!=\! n\alpha _n + o(n\tilde{\alpha }_n), \end{aligned}$$

and similarly, using also (2.15),

3.48 $$\begin{aligned} h_{\mathrm {I},n}''(0)&= 4 \sum _{k=0}^\infty kn_{k}- \sum _{k=0}^\infty k^3 n_{\mathrm {S},k}+ 3\frac{\rho _n}{\beta _n}\sum _{k=0}^\infty kn_{k}\nonumber \\&= 3(1 + \rho _n/\beta _n) \sum _{k=0}^\infty kn_{k}+ \sum _{k=0}^\infty kn_{\mathrm {S},k}- \sum _{k=0}^\infty k^3 n_{\mathrm {S},k}+ \sum _{k=0}^\infty k n_{\mathrm {I},k}\nonumber \\&= 3(1 + \rho _n/\beta _n) \sum _{k=0}^\infty kn_{k}- \sum _{k=0}^\infty k(k-1)(k-2 + 3)n_{\mathrm {S},k}+ \sum _{k=0}^\infty kn_{\mathrm {I},k}\nonumber \\&= -3n_{\mathrm {S}}\alpha _n - \sum _{k=0}^\infty k(k-1)(k-2)n_{\mathrm {S},k}+ O(n\tilde{\alpha }_n^2) =-n\lambda _3+o(n) . \end{aligned}$$

Also, for $$ t\geqslant 0$$,

$$\begin{aligned} |h_{\mathrm {I},n}'''(t)|&\leqslant (8 + 7\rho _n/\beta _n)\sum _{k=0}^\infty k n_{k}+ \sum _{k=0}^\infty k^4 n_{\mathrm {S},k}e^{-kt} = O(n) + \sum _{k=0}^\infty k^4 n_{\mathrm {S},k}e^{-kt}. \end{aligned}$$

Hence, for any $M \geqslant 1$,

$$\begin{aligned} \int _0^{\tilde{\alpha }_nt_0}|h_{\mathrm {I},n}'''(t)| \,\mathrm {d}t&\leqslant O(n\tilde{\alpha }_n) + \tilde{\alpha }_nt_0 \sum _{k \leqslant M} M^4n_{\mathrm {S},k}+ \sum _{k> M} k^3 n_{\mathrm {S},k}\bigl (1- e^{-k\tilde{\alpha }_nt_0}\bigr ) \\&\leqslant O(M^4 n\tilde{\alpha }_n) + \sum _{k> M} k^3 n_{\mathrm {S},k}= o(n) + \sum _{k > M} k^3 n_{\mathrm {S},k}. \end{aligned}$$

Letting $M \rightarrow \infty$ slowly (so that $M^4 \tilde{\alpha }_n=o(1)$), and using (D2), we obtain

3.49 $$\begin{aligned} \lim _{{n\rightarrow \infty }} \frac{1}{n} \int _0^{\tilde{\alpha }_nt_0}|h_{\mathrm {I},n}'''(t)| \,\mathrm {d}t = 0. \end{aligned}$$

Now, by a Taylor expansion, for $t\geqslant 0$,

3.50 $$\begin{aligned} h_{\mathrm {I},n}(\tilde{\alpha }_nt) = h_{\mathrm {I},n}(0) + h_{\mathrm {I},n}'(0)\tilde{\alpha }_nt + \tfrac{1}{2}h_{\mathrm {I},n}''(0)(\tilde{\alpha }_nt)^2 + \tfrac{1}{2}\int _0^{\tilde{\alpha }_nt}(\tilde{\alpha }_nt-u)^2h_{\mathrm {I},n}'''(u)\,\mathrm {d}u, \end{aligned}$$

and hence, using $h_{\mathrm {I},n}(0)=\sum _k kn_{\mathrm {I},k}$, (3.47), (3.48) and (3.49), uniformly in $t \leqslant t_0$,

3.51 $$\begin{aligned} h_{\mathrm {I},n}(\tilde{\alpha }_nt) = \sum _{k=0}^\infty kn_{\mathrm {I},k}+ n\alpha _n\tilde{\alpha }_nt - \tfrac{1}{2}\tilde{\alpha }_n^2 t^2 n\lambda _3+ o(n\tilde{\alpha }_n^2). \end{aligned}$$

If $\nu <\infty$, then $\tilde{\alpha }_n=\alpha _n$, and (3.51) yields by (2.8) and (3.3)

3.52 $$\begin{aligned} \begin{aligned} \frac{h_{\mathrm {I},n}(\tilde{\alpha }_nt)}{n\tilde{\alpha }_n^2} = \nu + t - \tfrac{1}{2} t^2 \lambda _3+ o(1) =f(t)+o(1). \end{aligned} \end{aligned}$$

If $\nu =\infty$, then (3.51) yields similarly by (3.35) and $\alpha _n=o(\tilde{\alpha }_n)$,

3.53 $$\begin{aligned} \begin{aligned} \frac{h_{\mathrm {I},n}(\tilde{\alpha }_nt)}{n\tilde{\alpha }_n^2} = 1 - \tfrac{1}{2} t^2 \lambda _3+ o(1) =f(t)+o(1). \end{aligned} \end{aligned}$$

Consequently, in both cases $h_{\mathrm {I},n}(\tilde{\alpha }_nt)/n\tilde{\alpha }_n^2 =f(t)+o(1),$ uniformly for $0\leqslant t\leqslant t_0$, and the result follows by combining this and (3.12) from Theorem 3.1. $\square$

We can now find (asymptotically) the duration $T^*$, except that when $\nu =0$, we cannot yet say whether the epidemic is very small or rather large.

#### Lemma 3.5

(i) If $$0<\nu \leqslant \infty $$, then $$T^*/\tilde{\alpha }_n\overset{\mathrm {p}}{\longrightarrow }\varkappa $$.
(ii) If $\nu =0$, then for every $\varepsilon > 0$, w.h.p., either
  - $$0 \leqslant T^*/\alpha _n < \varepsilon $$, or
  - $$|T^*/\alpha _n - \varkappa | < \varepsilon $$.

In particular, in both cases, w.h.p. $T^*\leqslant 2\varkappa \tilde{\alpha }_n$.

#### Proof

Take $t_0=2\varkappa$. Then $f(t_0)<0$, so (3.43) implies that $\mathbb {P}{}(T^*/\tilde{\alpha }_n\geqslant t_0)\rightarrow 0$, i.e., $T^*< t_0 \tilde{\alpha }_n$ w.h.p. Consequently, we may w.h.p. take $t=T^*/\tilde{\alpha }_n$ in (3.43) and conclude $\bigl |X_{\mathrm {I},T^*}/n\tilde{\alpha }_n^2 - f(T^*/\tilde{\alpha }_n)\bigr | \overset{\mathrm {p}}{\longrightarrow }0$. Since $X_{\mathrm {I},T^*}=0$ by definition, this says $f(T^*/\tilde{\alpha }_n) \overset{\mathrm {p}}{\longrightarrow }0$.

Consider *f*(*t*) for $t\in [0,\infty )$. If $\nu >0$, then *f*(*t*) has a unique zero at $\varkappa$, and is bounded away from 0 outside every neighbourhood of $\varkappa$; hence $T^*/\tilde{\alpha }_n\overset{\mathrm {p}}{\longrightarrow }\varkappa$ follows. If $\nu =0$, $f(t)=0$ both for $t=0$ and $t=\varkappa$, and (ii) follows. $\square$

#### Remark 3.6

Lemma 3.5 shows that taking $t_0 {:=} 2\varkappa$ in Theorem 3.1, we have w.h.p. $\tilde{\alpha }_nt_0\wedge T^*=T^*$, and thus, for $\tilde{\alpha }_n$ as above, Theorem 3.1 holds also with the suprema taken over all $t\leqslant T^*$.

### Final size

#### Proof of Theorem 2.4

Recall that ${\mathcal Z}_k {:=} n_{\mathrm {S},k}- S_{T^*}(k)$ is the number of susceptibles of degree *k* that ever become infected, and ${\mathcal Z}=\sum _k{\mathcal Z}_k$. For each $k \in {\mathbb Z}^+$,

3.54 $$\begin{aligned} \left| \frac{{\mathcal Z}_k}{n\tilde{\alpha }_n} - k p_k \frac{T^*}{\tilde{\alpha }_n} \right|&\leqslant \left| \frac{n_{\mathrm {S},k}(1- e^{-k T^*})}{n\tilde{\alpha }_n} - k p_k \frac{T^*}{\tilde{\alpha }_n}\right| + \left| \frac{S_{T^*}(k) - n_{\mathrm {S},k}e^{-k T^*}}{n\tilde{\alpha }_n}\right| . \end{aligned}$$

Since $|1 - e^{-y}-y| \leqslant y^2$ for all $y \geqslant 0$, and using (D1), (D2) and (3.3),

$$\begin{aligned} \sum _{k=0}^\infty \left| \frac{(1 - e^{-k \tilde{\alpha }_nt})n_{\mathrm {S},k}}{n\tilde{\alpha }_n} - kp_k t\right|&\leqslant t \sum _{k=0}^\infty k|p_k - n_{\mathrm {S},k}/n| + \tilde{\alpha }_nt^2\sum _{k=0}^\infty \frac{n_{\mathrm {S},k}k^2}{n} \rightarrow 0, \end{aligned}$$

uniformly in $t \leqslant t_0 {:=} 2\varkappa$. Since $T^*/\tilde{\alpha }_n\leqslant t_0$ w.h.p. by Lemma 3.5, it follows that

$$\begin{aligned} \sum _{k=0}^\infty \left| \frac{n_{\mathrm {S},k}(1- e^{-k T^*})}{n\tilde{\alpha }_n} - k p_k \frac{T^*}{\tilde{\alpha }_n}\right| = o_{\mathrm p}(1). \end{aligned}$$

Further, by (3.9) in Theorem 3.1 and Lemma 3.5, see Remark 3.6,

$$\begin{aligned} \sum _{k=0}^\infty \left| \frac{S_{T^*}(k) - n_{\mathrm {S},k}e^{-k T^*}}{n\tilde{\alpha }_n}\right| = o_{\mathrm p}(\tilde{\alpha }_n) = o_{\mathrm p}(1). \end{aligned}$$

It follows by (3.54) that

3.55 $$\begin{aligned} \sum _{k=0}^\infty \left| \frac{{\mathcal Z}_k}{n\tilde{\alpha }_n} - k p_k \frac{T^*}{\tilde{\alpha }_n} \right| = o_{\mathrm p}(1) \end{aligned}$$

and, in particular,

3.56 $$\begin{aligned} \left| \frac{{\mathcal Z}}{n\tilde{\alpha }_n} - \lambda \frac{T^*}{\tilde{\alpha }_n} \right| = \left| \sum _{k=0}^\infty \left( \frac{{\mathcal Z}_k}{n\tilde{\alpha }_n} - k p_k \frac{T^*}{\tilde{\alpha }_n}\right) \right| = o_{\mathrm p}(1). \end{aligned}$$

The estimate (3.56) and Lemma 3.5 yield the conclusions (i)–(iii) by (3.3) and the definitions of $\tilde{\alpha }_n$ and $\varkappa$ in (3.32)–(3.37). For (i), when $\nu =0$, we first obtain that if $\varepsilon _n=\varepsilon >0$ is fixed but arbitrary, then the conclusion holds w.h.p., and it is easy to see that this implies the same for some sequence $\varepsilon _n\rightarrow 0$. (Note also that if $\nu =0$, then $\varkappa =2/\lambda _3$.)

Furthermore, combining (3.55) and (3.56), we obtain

3.57 $$\begin{aligned} \sum _{k=0}^\infty \left| \frac{{\mathcal Z}_k}{n\tilde{\alpha }_n} - \frac{k p_k}{\lambda } \frac{{\mathcal Z}}{n\tilde{\alpha }_n} \right| = o_{\mathrm p}(1). \end{aligned}$$

We have shown that, except in the case (i)(a), there exists $\varepsilon >0$ such that w.h.p. ${\mathcal Z}/n\tilde{\alpha }_n\geqslant \varepsilon$; then (2.17) follows from (3.57). $\square$

## Proof of Theorem 2.5

We continue to use the simplifying assumptions in Sect. 3.1. We consider the epidemic in the original time scale and construct it from independent exponential random variables. At time $t=0$, we allocate each of the $n_{\mathrm {I}}$ initially infective vertices an ${\text {Exp}}(\rho _n)$ recovery time. We also give each free infective half-edge at time 0 an ${\text {Exp}}(\beta _n)$ pairing time. If the pairing time for a free infective half-edge is less than the recovery time of its parent vertex, then we colour that free half-edge red. Otherwise, we colour it black. We now wait until the first recovery or pairing time. At a recovery time, we change the status of the corresponding vertex to recovered. At a pairing time of a red free half-edge, we choose another free half-edge uniformly at random. If the chosen free half-edge belongs to a susceptible vertex then that vertex becomes infective, is given an ${\text {Exp}}(\rho _n)$ recovery time, and its remaining free half-edges are given independent ${\text {Exp}}(\beta _n)$ pairing times. Then, as above, we colour red any free half-edge with pairing time less than recovery time, and colour black all other free half-edges at the chosen vertex. The process continues in this fashion until no red free half-edges remain. Note that we do nothing at the pairing time of a black free half-edge, since it is no longer infective, and so black free half-edges behave like recovered free half-edges. Also, a red free half-edge will definitely initiate a pairing event at some point (provided it has not been chosen by another red free half-edge first). However, ignoring the colourings we obtain the same process as before.

Let $Z_t$ be the number of red free half-edges at time $t \geqslant 0$. Note that $Z_t$ changes only at pairing events, but not at recovery times. (The point of the colouring is to anticipate the recoveries, which then can be ignored.) Further, let $\bar{Z}_{m}:=Z_{T_m}$, where $T_m$ is the time of the *m*:th pairing event (and $T_0:=0$), and let $\zeta _m:=\Delta \bar{Z}_{m}:=\bar{Z}_{m}-\bar{Z}_{m-1}$. (Note that our processes are all right continuous, so $\bar{Z}_{m}$ is the number of red free half-edges immediately after the *m*-th pairing has occurred and we have coloured any new infective free half-edges.) Thus the process stops at $T_{m_*}$, where $m_*:=\min \{m\geqslant 0:\bar{Z}_{m}=0\}$. (This is not exactly the same stopping condition as used earlier, but the difference does not matter; there may still be some infective half-edges, but they are black and will recover before infecting any more vertex.) Let $\mathcal F_m =\mathcal F(T_m)$ be the corresponding discrete-time filtration generated by the coloured SIR process up to time $T_m$.

We keep the same notation as before for the free half-edge counts (so the total number of free infective half-edges, whether red or black, is $X_{\mathrm {I},t} \geqslant Z_t$, for example), and write again $S_t(k)$ for the number of susceptible vertices with *k* free half-edges at time $t \geqslant 0$. Furthermore, define

4.1 $$\begin{aligned} \pi _n:=\frac{\beta _n}{\beta _n+\rho _n}, \end{aligned}$$

the probability that a given free infective half-edge is coloured red. Note that $c\leqslant \pi _n\leqslant 1$ for some $c>0$ by (2.20).

We begin by showing that a substantial fraction of the initially infective half-edges are red. Recall that $X_{\mathrm {I},0}=\sum _{k=0}^\infty k n_{\mathrm {I},k}$ is the total degree of the initially infective vertices and that $d_{\mathrm {I},*}$ is the maximum degree among these vertices.

### Lemma 4.1

Suppose that $$X_{\mathrm {I},0} \rightarrow \infty $$.

(i) If $$d_{\mathrm {I},*}=o(X_{\mathrm {I},0})$$, then $$Z_0=\pi _n X_{\mathrm {I},0}\bigl (1+o_{\mathrm p}(1)\bigr )$$.
(ii) More generally, for any $d_{\mathrm {I},*}$, we have
  4.2 $$\begin{aligned} \lim _{\delta \rightarrow 0} \limsup _{n\rightarrow \infty } \mathbb {P}\Bigl (Z_0 \leqslant \delta X_{\mathrm {I},0}\Bigr ) = 0. \end{aligned}$$

### Proof

We enumerate all initially infective vertices as $i = 1, \ldots , n_{\mathrm {I}}$, and let $d_{\mathrm {I},i}$ be the degree of vertex *i*, so that $X_{\mathrm {I},0} = \sum _{i = 1}^{n_{\mathrm {I}}} d_{\mathrm {I},i}$. We also let $Z_{0,i}$ be the number of red free half-edges at vertex *i*, so $Z_0 = \sum _{i = 1}^{n_{\mathrm {I}}} Z_{0,i}$, where the $Z_{0,i}$ are independent, with $\mathbb {E}{}Z_{0,i} = d_{\mathrm {I},i}\pi _n$ and $Z_{0,i} \leqslant d_{\mathrm {I},i}$. It follows that $\mathbb {E}{}Z_0=\sum _{i = 1}^{n_{\mathrm {I}}}\mathbb {E}{}Z_{0,i} =\pi _nX_{\mathrm {I},0}$ and

4.3 $$\begin{aligned} {\text {Var}}Z_0 = \sum _{i = 1}^{n_{\mathrm {I}}} {\text {Var}}Z_{0,i} \leqslant \sum _{i = 1}^{n_{\mathrm {I}}} d_{\mathrm {I},i}^2 \leqslant d_{\mathrm {I},*}X_{\mathrm {I},0}. \end{aligned}$$

(i) If $d_{\mathrm {I},*}=o(X_{\mathrm {I},0})$, then (4.3) yields ${\text {Var}}Z_0=o(X_{\mathrm {I},0}^2)=o((\mathbb {E}{}Z_0)^2)$, and thus Chebyshev’s inequality yields $Z_0=\mathbb {E}{}Z_0(1+o_{\mathrm p}(1))$.

(ii) Take any $\delta > 0$ with $\delta <\frac{1}{2}\min _n \pi _n$.

We assume first that $d_{\mathrm {I},*}\leqslant \delta ^{1/2} X_{\mathrm {I},0}$. Then (4.3) and Chebyshev’s inequality yield

4.4 $$\begin{aligned} \mathbb {P}{}(Z_0\leqslant \delta X_{\mathrm {I},0}) \!\leqslant \!\frac{{\text {Var}}Z_0}{(\mathbb {E}{}Z_0-\delta X_{\mathrm {I},0})^2} \!\leqslant \! \frac{d_{\mathrm {I},*}X_{\mathrm {I},0}}{(\frac{1}{2}\pi _nX_{\mathrm {I},0})^2} \!\leqslant \!4\pi _n^{-2}\delta ^{1/2}\!=\! 4\left( \frac{\rho _n+\beta _n}{\beta _n}\right) ^{2}\delta ^{1/2}. \end{aligned}$$

Assume now instead that $d_{\mathrm {I},*}\geqslant \delta ^{1/2} X_{\mathrm {I},0}$. Fix one initially infective vertex of degree $d_{\mathrm {I},*}$, let $Z_{0,*}$ be the number of red free half-edges at that vertex, and let $R_\star$ be its recovery time. Then $Z_0 \geqslant Z_{0,*}$, and so $Z_0 \leqslant \delta X_{\mathrm {I},0}$ implies that $Z_{0,*}\leqslant \delta ^{1/2} d_{\mathrm {I},*}$. We have

4.5 $$\begin{aligned} \mathbb {P}(Z_{0,*}\leqslant \delta ^{1/2} d_{\mathrm {I},*})&= \mathbb {P}(Z_{0,*}\leqslant \delta ^{1/2}d_{\mathrm {I},*}, R_\star \leqslant 4\delta ^{1/2}/\beta _n ) \nonumber \\&\quad + \mathbb {P}(Z_{0,*}\leqslant \delta ^{1/2} d_{\mathrm {I},*}, R_\star> 4\delta ^{1/2}/\beta _n )\leqslant \mathbb {P}( R_\star \leqslant 4\delta ^{1/2}/\beta _n ) \nonumber \\&\quad + \mathbb {P}\bigl (Z_{0,*}\leqslant \delta ^{1/2}d_{\mathrm {I},*}\mid R_\star > 4\delta ^{1/2}/\beta _n\bigr ). \end{aligned}$$

Now,

4.6 $$\begin{aligned} \mathbb {P}( R_\star \leqslant 4\delta ^{1/2}/\beta _n ) = 1 - e^{-4\delta ^{1/2}\rho _n/\beta _n} \leqslant 4\delta ^{1/2}\rho _n/\beta _n. \end{aligned}$$

Also, conditional on $R_\star = r$, $Z_{0,*}$ has a binomial distribution with parameters $d_{\mathrm {I},*}$ and $1-e^{-\beta _n r}$. It follows that, conditional on $R_\star > 4\delta ^{1/2}/\beta _n$, $Z_{0,*}$ stochastically dominates a ${\text {Bin}}(d_{\mathrm {I},*}, 1 - e^{-4\delta ^{1/2}} )$ random variable. For $\delta$ small enough, $1 - e^{-4\delta ^{1/2}} \geqslant 2 \delta ^{1/2}$, and so, by Chebyshev’s inequality,

4.7 $$\begin{aligned} \mathbb {P}\left( Z_{0,*}\leqslant \delta ^{1/2} d_{\mathrm {I},*}\mid R_\star > \frac{4\delta ^{1/2}}{\beta _n}\right)&\leqslant \mathbb {P}\Bigl ({\text {Bin}}(d_{\mathrm {I},*}, 2\delta ^{1/2}) \leqslant \delta ^{1/2} d_{\mathrm {I},*}\Bigr )\nonumber \\&\leqslant \frac{2\delta ^{1/2}d_{\mathrm {I},*}}{\delta d_{\mathrm {I},*}^2} \leqslant \frac{2}{\delta X_{\mathrm {I},0}}. \end{aligned}$$

Combining (4.4), (4.5), (4.6) and (4.7), we see that if $\delta$ is small, then in both cases

$$\begin{aligned} \mathbb {P}\bigl (Z_0 \leqslant \delta X_{\mathrm {I},0} \bigr ) \leqslant 4\left( \frac{\rho _n+\beta _n}{\beta _n}\right) ^{2}\delta ^{1/2}+ \frac{2}{\delta X_{\mathrm {I},0}}, \end{aligned}$$

and (4.2) follows, since $X_{\mathrm {I},0}\rightarrow \infty$ and $\rho _n/\beta _n=O(1)$ by (2.20). $\square$

### Lemma 4.2

Let $(W_m)_{m=0}^\infty$ be a process adapted to a filtration $(\mathcal F_m)_{m = 0}^\infty$, with $W_0 = 0$, and let $\tau \leqslant \infty$ be a stopping time. Suppose that the positive numbers $v,w > 0$ are such that

4.8 $$\begin{aligned} \mathbb {E}{}[ \Delta W_{m+1}\mid \mathcal F_m]&\geqslant v \quad \text {a.s.~on } \{m<\tau \}, \end{aligned}$$

4.9 $$\begin{aligned} \mathbb {E}{}[(\Delta W_{m+1})^2]&\leqslant w \end{aligned}$$

for every $m \geqslant 0$. Then, for any $b>0$,

4.10 $$\begin{aligned} \mathbb {P}\left( \inf _{0\leqslant m \leqslant \tau } W_m \leqslant -b\right) \leqslant \frac{8 w}{bv}. \end{aligned}$$

### Proof

Consider the Doob decomposition

4.11 $$\begin{aligned} W_m = M_m + A_m, \end{aligned}$$

where $A_m {:=} \sum _{l=1}^m \mathbb {E}{}[\Delta W_{l}\mid \mathcal F_{l-1}]$ is predictable and $M_m {:=} W_m - A_m$ is a martingale with respect to $(\mathcal F_m)$. By the assumption (4.8), $A_m\geqslant mv$ a.s. when $m\leqslant \tau$. Furthermore,

$$\begin{aligned} \mathbb {E}{}[\Delta M_m^2]&= \mathbb {E}{}[(\Delta W_m - \mathbb {E}{}[\Delta W_m \mid \mathcal F_{m-1}] )^2] = \mathbb {E}{}[(\Delta W_m)^2]\\&\quad - \mathbb {E}{}(\mathbb {E}{}[\Delta W_m \mid \mathcal F_{m-1}])^2 \leqslant w. \end{aligned}$$

Thus, by Doob’s inequality, for any $N\geqslant 1$,

4.12 $$\begin{aligned} \mathbb {P}\left( \inf _{N\leqslant m \leqslant (2N) \wedge \tau } W_m \leqslant -b\right)\leqslant & {} \mathbb {P}\left( \inf _{N\leqslant m \leqslant 2N} M_m \leqslant -b-Nv\right) \nonumber \\\leqslant & {} \frac{\mathbb {E}{}[M_{2N}^2]}{(b+Nv)^2} \leqslant \frac{2N w}{(b+Nv)^2}. \end{aligned}$$

Summing over all powers of 2, we obtain

$$\begin{aligned} \mathbb {P}\left( \inf _{1\leqslant m \leqslant \tau } W_m \leqslant -b\right)\leqslant & {} \sum _{k=0}^\infty \mathbb {P}\left( \inf _{2^k\leqslant m \leqslant 2^{k+1}\wedge \tau } W_m \leqslant -b\right) \leqslant \sum _{k=0}^\infty \frac{2^{k+1} w}{(b+2^kv)^2}\\\leqslant & {} \sum _{2^k \leqslant b/v} \frac{2^{k+1} w}{b^2} + \sum _{2^k \geqslant b/v} \frac{2^{k+1} w}{2^{2k}v^2} \leqslant \frac{4(b/v)w}{b^2}\\&+\frac{4(v/b)w}{v^2}=\frac{8w}{bv}. \end{aligned}$$

$$\square $$

### Proof of Theorem 2.5(ii)

Let $$\delta > 0$$ be a small positive number chosen later. Define the discrete stopping time $$m_{**}$$ by

4.13 $$\begin{aligned} m_{**}:=\min \left\{ m\geqslant 0: \bar{Z}_{m}=0 \quad \text {or}\quad \sum _{k=0}^\infty k^2 (n_{\mathrm {S},k}- S_{T_m}(k)) > \delta n \alpha _n\right\} . \end{aligned}$$

Note that for $$m<m_{**}$$, the total number of free half-edges at time $$T_m$$ is

4.14 $$\begin{aligned} \begin{aligned} X_{T_m} \geqslant \sum _{k=0}^\infty kS_{T_m}(k) \geqslant \sum _{k=0}^\infty kn_{\mathrm {S},k}- \delta n\alpha _n \geqslant \sum _{k=0}^\infty kn_{k}- \delta n\alpha _n -o(n\alpha _n), \end{aligned} \end{aligned}$$

since $$\sum _{k=0}^\infty kn_{\mathrm {I},k}=o(n_{\mathrm {S}}\alpha _n^2)=o(n\alpha _n)$$ by (2.6) and (2.8). Similarly, for $$m<m_{**}$$,

4.15 $$\begin{aligned} \bar{Z}_{m}=Z_{T_m} \leqslant Z_0 + \sum _{k=0}^\infty k (n_{\mathrm {S},k}- S_{T_m}(k)) \leqslant Z_0 + \delta n\alpha _n \leqslant \delta n\alpha _n + o(n\alpha _n), \end{aligned}$$

since $Z_0$ is bounded above by $\sum _{k=0}^\infty kn_{\mathrm {I},k}= o(n_{\mathrm {S}}\alpha _n^2) = o(n\alpha _n)$.

At a pairing $$m +1 \leqslant m_{**}$$, a red free half-edge pairs with a free susceptible half-edge, or with another red free half-edge, or with a black half-edge. In the first case, if the susceptible half-edge belongs to a vertex of degree *k*, we get on the average $$\pi _n (k-1)$$ new red free half-edges; in the second case we instead lose one red free half-edge, in addition to the pairing red free half-edge that we always lose. The probability of pairing with a susceptible half-edge belonging to a vertex of degree *k* is $$kS_{T_m}(k)/X_{T_m}$$ and the probability of pairing with another red free half-edge is $$\bar{Z}_{m}/X_{T_m}$$. Hence, for $$m+1 \leqslant m_{**}$$, using (4.13)–(4.15), (4.1) and the definition (2.1) of $${\mathcal R}_0$$,

$$\begin{aligned} \mathbb {E}{}[\Delta \bar{Z}_{m+1}\mid \mathcal F_m]&\geqslant -1 + \pi _n \frac{\sum _{k=0}^\infty (k-1)k S_{T_m}(k)}{\sum _{k=0}^\infty kn_{k}} - \frac{\bar{Z}_{m}}{\sum _{k=0}^\infty kn_{k}- (\delta +o(1)) n \alpha _n}\\&\geqslant -1 + \pi _n \frac{\sum _{k=0}^\infty (k-1)kn_{\mathrm {S},k}-\delta n\alpha _n}{\sum _{k=0}^\infty kn_{k}} -\frac{(\delta +o(1))n\alpha _n}{\sum _{k=0}^\infty kn_{k}- (\delta +o(1)) n \alpha _n}\\&\geqslant -1 + \mathcal {R}_0- O(\delta \alpha _n). \end{aligned}$$

Since $$(\mathcal {R}_0-1)\alpha _n^{-1}$$ is bounded away from 0 by (2.3) and Remark 2.9, this shows that as long as $$\delta $$ is chosen small enough there exists some $${}c_{1}> 0$$ such that if *n* is large and $$m< m_{**}$$, then

4.16 $$\begin{aligned} \mathbb {E}{}[\Delta \bar{Z}_{m+1}\mid \mathcal F_m] \geqslant c_{1}\alpha _n. \end{aligned}$$

Furthermore, noting that the number of red free half-edges may change by at most *k* at a jump if a red free half-edge pairs with a free susceptible half-edge at a vertex of degree *k*, the expected square of any jump satisfies, for $$m<m_{**}$$,

4.17 $$\begin{aligned} \mathbb {E}{}[ (\Delta \bar{Z}_{m+1})^2\mid \mathcal F_m ] \leqslant 4 + \frac{\sum _{k=0}^\infty k^3n_{\mathrm {S},k}}{\sum _{k=0}^\infty kn_{k}-(\delta +o(1))n\alpha _n} \leqslant {}c_{2}, \end{aligned}$$

for some $c_{2}> 0$, uniformly in all large *n*, by assumption (D2).

Let $$W_m = \bar{Z}_{m\wedge m_{**}}-Z_0$$. It follows from (4.16) and (4.17) that Lemma 4.2 applies with $$\tau =m_{**}$$, $$v=c_{1}\alpha _n$$ and $$w=c_{2}$$.

Let $a=a_n> 0$ satisfy $a_n \rightarrow \infty$ and $a_n =o\bigl (\alpha _n \sum _{k=0}^\infty k n_{\mathrm {I},k}\bigr )$ as $n \rightarrow \infty$. Then Lemma 4.2 with $b=a_n/\alpha _n$ yields

4.18 $$\begin{aligned} \mathbb {P}\left( \inf _{0\leqslant m \leqslant m_{**}} W_m \leqslant -a_n/\alpha _n\right) \leqslant \frac{8 c_{2}}{c_{1}a_n} =o(1) \end{aligned}$$

and thus $$W_{m_{**}}>-a_n/\alpha _n$$ w.h.p. On the other hand, Lemma 4.1(ii) implies that $$\mathbb {P}{}(Z_0\leqslant a_n/\alpha _n)\rightarrow 0$$. Consequently, $$\bar{Z}_{m_{**}}=W_{m_{**}}+Z_0>0$$ w.h.p. By (4.13), this means that w.h.p. $$\sum _{k=0}^\infty k^2 (n_{\mathrm {S},k}- S_{T_{m_{**}}}(k)) > \delta n \alpha _n$$, and thus that, for some time *t* before the epidemic dies out, $$\sum _{k=0}^\infty k^2 (n_{\mathrm {S},k}- S_{t}(k)) > \delta n \alpha _n$$.

The latter statement does not depend on the time-scale, so it holds for the time-changed epidemic in Sect. 3.2 too. Thus, using the notation there, by the monotonicity of the number of susceptibles, w.h.p.

4.19 $$\begin{aligned} \sum _{k=0}^\infty k^2 (n_{\mathrm {S},k}- S_{T^*}(k)) > \delta n \alpha _n. \end{aligned}$$

On the other hand, Lemma 3.2 (with $\tilde{\alpha }_n=\alpha _n$) and (2.10) yield, for any $\varepsilon >0$,

4.20 $$\begin{aligned} \sup _{0 \leqslant t \leqslant \varepsilon \alpha _n \wedge T^*}\sum _{k=0}^\infty k^2(n_{\mathrm {S},k}- S_{t}(k))\leqslant & {} \sup _{t\leqslant \varepsilon \alpha _n \wedge T^*} \left| \sum _{k=0}^\infty k^2(S_{t}(k) - n_{\mathrm {S},k}e^{-k t})\right| \nonumber \\&+ \varepsilon \alpha _n \sum _{k=0}^\infty k^3 n_{\mathrm {S},k}\leqslant o_{\mathrm p}(n \alpha _n)+ \varepsilon c_0 n \alpha _n. \end{aligned}$$

Consequently, if we first choose $\delta >0$ so small that (4.19) holds, and then $\varepsilon <\delta /c_0$, then (4.19) and (4.20) imply that w.h.p. $T^*>\varepsilon \alpha _n$. It follows by Lemma 3.5 that $T^*/\alpha _n \overset{\mathrm {p}}{\longrightarrow }\varkappa =2/\lambda _3$, and thus the result follows by (3.56). $\square$

To study the cases (i) and (iii), we analyse the number of red free half-edges more carefully. Let the random variable *Y*(*k*) be the number of new red free half-edges when a vertex of degree *k* is infected. Given the recovery time $\tau$ of the vertex, $Y(k)\sim {\text {Bin}}\bigl (k-1, 1-e^{-\beta _n\tau }\bigr )$, and, since $\tau \sim {\text {Exp}}(\rho _n)$, the probability $1-e^{-\beta _n\tau }$ has the Beta distribution $B(1,\rho _n/\beta _n)$. Consequently, *Y*(*k*) has the beta-binomial distribution with parameters $(k-1,1,\rho _n/\beta _n)$. More generally, if *D* is a positive integer valued random variable, then *Y*(*D*) denotes a random variable that conditioned on $D=k$ has the distribution *Y*(*k*). We have the following elementary result, recalling the notation (4.1).

### Lemma 4.3

For any positive integer valued random variable *D*,

4.21 $$\begin{aligned} \mathbb {E}{}Y(D)&= \pi _n(\mathbb {E}{}D-1), \end{aligned}$$

4.22 $$\begin{aligned} \mathbb {E}{}Y(D)^2&=\frac{\pi _n^2\mathbb {E}{}(D-1)(2D-3)+\pi _n\mathbb {E}{}(D-1)}{1+\pi _n}. \end{aligned}$$

### Proof

For each $k\geqslant 1$, we obtain by conditioning on the recovery time $\tau$,

4.23 $$\begin{aligned} \mathbb {E}{}Y(k)&= (k-1) \frac{1}{1+\rho _n/\beta _n}=(k-1)\pi _n, \end{aligned}$$

4.24 $$\begin{aligned} \mathbb {E}{}Y(k)^2&= \frac{(k-1)(2k-2+\rho _n/\beta _n)}{(1+\rho _n/\beta _n)(2+\rho _n/\beta _n)} =\frac{(k-1)(2k-3)\pi _n^2+(k-1)\pi _n}{1+\pi _n} \end{aligned}$$

and (4.21) and (4.22) follows by conditioning on *D*. $\square$

Let *A* be a constant, and consider only $m\leqslant M:=\lfloor A\alpha _n^{-2}\rfloor$. At pairing event *m* for $m \leqslant M$, the number of free half-edges is at least $\sum _k kn_{k}-2A\alpha _n^{-2}\geqslant \sum _k kn_{k}\cdot (1-A_1 n^{-1}\alpha _n^{-2})$ for some constant $A_1$. Thus, the probability that a susceptible vertex with $\ell$ half-edges is infected is at most, for $A_2:=2A_1$ and large *n*,

4.25 $$\begin{aligned} \frac{\ell n_{\mathrm {S},\ell }}{\sum _k kn_{k}\cdot (1-A_1 n^{-1}\alpha _n^{-2})} \leqslant \bigl (1+A_2 n^{-1}\alpha _n^{-2}\bigr ) \frac{\ell n_{\mathrm {S},\ell }}{\sum _k kn_{k}}. \end{aligned}$$

Let $D^+\geqslant 1$ be a random variable with the distribution

4.26 $$\begin{aligned} \mathbb {P}{}(D^+\geqslant j) := \min \left( \bigl (1+A_2 n^{-1}\alpha _n^{-2}\bigr )\frac{\sum _{k\geqslant j}k n_{\mathrm {S},k}}{\sum _k kn_{k}} ,1\right) , \quad j\geqslant 2, \end{aligned}$$

and let $\zeta ^+:=Y(D^+)-1$. (Note that $D^+$ and $\zeta ^+$ depend on *n*, although we omit this from the notation.) Then $\zeta _m:=\Delta \bar{Z}_{m}$, conditioned on what has happened earlier, is stochastically dominated by $\zeta ^+$. Hence, there exist independent copies $(\zeta ^+_m)_1^\infty$ of $\zeta ^+$ such that $\zeta _m\leqslant \zeta ^+_m$ for all $m\leqslant M$ such that the epidemic has not yet stopped; furthermore, $(\zeta ^+_m)_1^\infty$ are also independent of $Z_0$. If the epidemic stops at $m_*<M$ (because $Z_{m_*}=0$ so there are no more pairing events), then we for convenience extend the definition of $\zeta _m$ and $\bar{Z}_{m}$ to all $m\leqslant M$ by defining $\zeta _m:=\zeta ^+_m$ for $m>m_*$, and still requiring $\zeta _m=\Delta \bar{Z}_{m}$. Consequently, $\zeta _m\leqslant \zeta _m^+$ for all $m\leqslant M$ and thus the (possibly extended) sequence $(\bar{Z}_{m})_0^M$ is dominated by the random walk $(\bar{Z}_{m}^+)_0^M$ with $\bar{Z}_{m}^+:=Z_0+\sum _{i=1}^m \zeta ^+_i$.

Next, observe that (4.26) implies

4.27 $$\begin{aligned} \mathbb {E}{}D^+-1 = \sum _{j=2}^\infty \mathbb {P}{}(D^+\geqslant j) \geqslant \sum _{j=2}^\infty \frac{\sum _{k\geqslant j}k n_{\mathrm {S},k}}{\sum _k kn_{k}} = \frac{\sum _{k} (k-1)kn_{\mathrm {S},k}}{\sum _k kn_{k}} \end{aligned}$$

and also, since $n\alpha _n^3\rightarrow \infty$,

4.28 $$\begin{aligned} \sum _{j=2}^\infty \mathbb {P}{}(D^+\geqslant j) \leqslant \sum _{j=2}^\infty \bigl (1+A_2 n^{-1}\alpha _n^{-2}\bigr )\frac{\sum _{k\geqslant j}k n_{\mathrm {S},k}}{\sum _k kn_{k}} =\bigl (1+o(\alpha _n)\bigr ) \frac{\sum _{k} (k-1)kn_{\mathrm {S},k}}{\sum _k kn_{k}}. \end{aligned}$$

It thus follows that

4.29 $$\begin{aligned} \mathbb {E}{}D^+-1 = \sum _{j=2}^\infty \mathbb {P}{}(D^+\geqslant j) =\bigl (1+o(\alpha _n)\bigr ) \frac{\sum _{k} (k-1)kn_{\mathrm {S},k}}{\sum _k kn_{k}}. \end{aligned}$$

Hence, using (4.21), (4.1), (2.1) and (2.22),

4.30 $$\begin{aligned} \begin{aligned} \mathbb {E}{}\zeta ^+&= \frac{\beta _n}{\beta _n+\rho _n}\mathbb {E}{}(D^+-1)-1 = \bigl (1+o(\alpha _n)\bigr )\frac{\beta _n}{\beta _n+\rho _n} \frac{\sum _{k} (k-1)kn_{\mathrm {S},k}}{\sum _k kn_{k}} -1\\&=\bigl (1+o(\alpha _n)\bigr ){\mathcal R}_0-1 ={\mathcal R}_0-1+o(\alpha _n) =\lambda _2^{-1}\alpha _n+o(\alpha _n). \end{aligned} \end{aligned}$$

Note also that (4.26), using (2.4), (2.6), (2.7), shows that as ${n\rightarrow \infty }$, $D^+\overset{\mathrm {d}}{\longrightarrow }\hat{D}_{\mathrm {S}}$, where $\hat{D}_{\mathrm {S}}$ has the size-biased distribution $\mathbb {P}{}(\hat{D}_{\mathrm {S}}=k)=kp_k/\lambda$. Moreover, it follows easily from (D2) and (4.26) that $D^+$ is uniformly square integrable as ${n\rightarrow \infty }$, and thus

4.31 $$\begin{aligned} \begin{aligned} \mathbb {E}{}(D^+)^2\rightarrow \mathbb {E}{}(\hat{D}_{\mathrm {S}})^2 =\frac{\sum _{k=0}^\infty k^3p_k}{\sum _{k=0}^\infty kp_k}=\frac{\mathbb {E}{}D_{\mathrm {S}}^3}{\mathbb {E}{}D_{\mathrm {S}}} \end{aligned} \end{aligned}$$

and similarly

4.32 $$\begin{aligned} \begin{aligned} \mathbb {E}{}D^+\rightarrow \mathbb {E}{}\hat{D}_{\mathrm {S}}=\frac{\mathbb {E}{}D_{\mathrm {S}}^2}{\mathbb {E}{}D_{\mathrm {S}}}. \end{aligned} \end{aligned}$$

By (4.30) and $\alpha _n\rightarrow 0$ we have $\mathbb {E}{}\zeta ^+\rightarrow 0$, and thus $\pi _n(\mathbb {E}{}D^+-1)\rightarrow 1$. Hence, by (4.32) (or directly from (2.1)),

4.33 $$\begin{aligned} \pi _n =\frac{\beta _n}{\beta _n+\rho _n}\rightarrow \frac{\mathbb {E}{}D_{\mathrm {S}}}{\mathbb {E}{}D_{\mathrm {S}}(D_{\mathrm {S}}-1)} =\frac{\lambda }{\lambda _2}. \end{aligned}$$

Since $|\zeta ^+|\leqslant D^+$, it follows that also $\zeta ^+$ is uniformly square integrable as ${n\rightarrow \infty }$. Furthermore, $\mathbb {E}{}Y(D^+) = 1+\mathbb {E}{}\zeta ^+=1+o(1)$ and thus, using (4.22) and (4.31)–(4.33),

4.34 $$\begin{aligned} {\text {Var}}\zeta ^+= & {} {\text {Var}}(Y(D^+))= \mathbb {E}{}(Y(D^+)^2)-\bigl (1+o(1)\bigr )^2 \nonumber \\= & {} \frac{\pi _n^2}{1+\pi _n}\mathbb {E}{}(D^+-1)(2D^+-3) + \frac{\pi _n}{1+\pi _n}\mathbb {E}{}(D^+-1) -1+o(1) \nonumber \\&\rightarrow \frac{\lambda ^2}{\lambda _2(\lambda _2+\lambda )}\frac{\mathbb {E}{}D_{\mathrm {S}}(D_{\mathrm {S}}-1)(2D_{\mathrm {S}}-3)}{\mathbb {E}{}D_{\mathrm {S}}} + \frac{\lambda }{\lambda _2+\lambda }\frac{\mathbb {E}{}D_{\mathrm {S}}(D_{\mathrm {S}}-1)}{\mathbb {E}{}D_{\mathrm {S}}} -1 \nonumber \\= & {} \frac{\lambda (2\lambda _3+\lambda _2)}{\lambda _2(\lambda _2+\lambda )} + \frac{\lambda _2}{\lambda _2+\lambda } -1 \nonumber \\= & {} \frac{2\lambda \lambda _3}{\lambda _2(\lambda _2+\lambda )} =:\sigma ^2. \end{aligned}$$

Now consider $\alpha _n(\bar{Z}_{M}^+ -Z_0-M\mathbb {E}{}\zeta ^+) =\alpha _n\sum _{i=1}^{\lfloor A\alpha _n^{-2}\rfloor }(\zeta ^+_i-\mathbb {E}{}\zeta ^+)$. The summands are i.i.d. with mean 0, and the uniform square integrability of $\zeta ^+$ implies that the Lindeberg condition holds; thus the central limit theorem (Kallenberg 2002, Theorem 5.12) applies and yields, using (4.34), $\alpha _n(\bar{Z}_{M}^+ -Z_0-M\mathbb {E}{}\zeta ^+)\overset{\mathrm {d}}{\longrightarrow }N(0,A\sigma ^2)$ as ${n\rightarrow \infty }$. Moreover, normal convergence of the endpoint of a random walk implies Donsker-type convergence of the entire random walk to a Brownian motion, see Kallenberg (2002, Theorem 14.20); hence,

4.35 $$\begin{aligned} \alpha _n\left( \bar{Z}_{t\alpha _n^{-2}}^+ -Z_0-t\alpha _n^{-2}\mathbb {E}{}\zeta ^+\right) \rightarrow \sigma B_t, \end{aligned}$$

where $B_t$ is a standard Brownian motion and we have defined $\bar{Z}_{t}^+$ also for non-integer *t* by $\bar{Z}_{t}^+:=\bar{Z}_{\lfloor t\rfloor }^+$. (We define $\bar{Z}_{t}$ and $\bar{Z}_{t}^-$ below in the same way.) Here the convergence is in distribution in the Skorohod space *D*[0, *A*], but we may by the Skorohod coupling theorem (Kallenberg 2002, Theorem 4.30) assume that the processes for different *n* are coupled such that a.s. (4.35) holds uniformly on [0, *A*].

Moreover, $\alpha _n^{-1}\mathbb {E}{}\zeta ^+\rightarrow \lambda _2^{-1}$ by (4.30), and thus (4.35) implies

4.36 $$\begin{aligned} \alpha _n\left( \bar{Z}_{t\alpha _n^{-2}}^+ -Z_0\right) \rightarrow \sigma B_t + \lambda _2^{-1}t . \end{aligned}$$

### Proof of Theorem 2.5(i)

In this case, $\alpha _nX_{\mathrm {I},0}\rightarrow 0$, and, since $0\leqslant Z_0\leqslant X_{\mathrm {I},0}$, it follows from (4.36) that $\alpha _n\bar{Z}_{t\alpha _n^{-2}}^+ \rightarrow \sigma B_t + \lambda _2^{-1}t$, where (as said above) we may assume that the convergence holds uniformly on [0, *A*] a.s. For any fixed $\delta >0$, the right-hand side is a.s. negative for some $t\in [0,\delta ]$, and thus w.h.p. $\alpha _n\bar{Z}_{t\alpha _n^{-2}}^+ <0$ for some $t\in [0,\delta ]$. Since $Z_m\leqslant \bar{Z}_{m}$ it follows that w.h.p. $m_*\leqslant \delta \alpha _n^{-2}$, i.e. the epidemic stops with $Z_m=0$ after at most $\delta \alpha _n^{-2}$ infections. Hence, w.h.p.

4.37 $$\begin{aligned} {\mathcal Z}\leqslant m_*\leqslant \delta \alpha _n^{-2}=o\bigl (n\alpha _n\bigr ). \end{aligned}$$

Since $\delta$ is arbitrary, this moreover shows ${\mathcal Z}=o_{\mathrm p}\bigl (\alpha _n^{-2}\bigr )$. $\square$

### Proof of Theorem 2.5(iii) in the multigraph case

We combine the upper bound $\bar{Z}_{m}^+$ above with a matching lower bound. Let $x_1,x_2,\ldots$ be an i.i.d. sequence of random half-edges, constructed before we run the epidemic by drawing with replacement from the set of all half-edges. Then, at the *m*:th pairing event, when we are to pair an infective red half-edge $y_m$, if $x_m$ still is free and $x_m\ne y_m$, we pair $y_m$ with $x_m$; otherwise we resample and pair $y_m$ with a uniformly chosen free half-edge $\ne y_m$. Furthermore, we let $\zeta _m^-:=-1$ if $x_m$ is initially infective, and if $x_m$ belongs to an initially susceptible vertex of degree *k*, we let $\zeta _m^-$ be a copy of $Y(k)-1$ (independent of the history); if $x_m$ still is susceptible at the *m*:th pairing event (and thus free, so we pair with $x_m$), we may assume that $\zeta _m^-:=\zeta _m$, the number of new red free half-edges minus 1. Note that $(\zeta _m^-)_{m\geqslant 1}$ is an i.i.d. sequence of random variables with the distribution $Y(D^-)-1$, where $D^-$ has the distribution obtained by taking $A_2=0$ in (4.26); furthermore, $(\zeta ^-_m)_1^\infty$ are independent of $Z_0$. Let $\bar{Z}_{m}^-:=Z_0+\sum _{i=1}^m\zeta _i^-$. Note that (4.27)–(4.34) hold for $D^-$ and $\zeta _m^-$ too (with some simplifications), and thus, in analogy with (4.36),

4.38 $$\begin{aligned} \alpha _n\left( \bar{Z}_{t\alpha _n^{-2}}^- -Z_0\right) \rightarrow \sigma B^-_t + \lambda _2^{-1}t , \end{aligned}$$

for some Brownian motion $B_t^-$. We next verify that we can take the same Brownian motion in (4.36) and (4.38).

Let $\zeta _m':=\zeta _m^--\zeta _m$. Thus $\zeta _m'=0$ if $x_m$ is susceptible at time $T_m$. If $x_m$ was initially susceptible, with degree *k*, but has been infected, then $\zeta _m'\leqslant \zeta _m^-+2\leqslant k$. If $x_m$ was initially infected, then $\zeta _m^-=-1$ and thus $\zeta _m'\leqslant \zeta _m^-+2\leqslant 1$.

Consider as above only $m\leqslant M:=\lfloor A\alpha _n^{-2}\rfloor$, for some (large) constant $A>0$. For $m>m_*$, when the epidemic has stopped, we have defined $\zeta _m=\zeta _m^+$. Since $\zeta _m^\pm \overset{\mathrm {d}}{=}Y(D^\pm )-1$ and $D^-$ is stochastically dominated by $D^+$, we may in this case assume that $\zeta _m=\zeta _m^+\geqslant \zeta _m^-$, and thus $\zeta _m'\leqslant 0$.

For $m\leqslant M$, the number of initially susceptible half-edges that have been infected is at most, using (2.11) and (2.6), $m d_{\mathrm {S},*}= O\bigl (\alpha _n^{-2}d_{\mathrm {S},*}\bigr ) = o\bigl (\alpha _n^{-2}n^{1/3}\bigr )=o(n)$. Hence the number of free half-edges at $T_m$ is at least $\sum _k kn_{\mathrm {S},k}-m d_{\mathrm {S},*}=\lambda n-o(n)\geqslant {}c_{3}n$ for $c_{3}:=\lambda /2$ if *n* is large enough. It follows that the probability that a given initially susceptible vertex of degree *k* has been infected before $T_m$ is at most $mk/(c_{3}n)$, and the probability that one of its half-edges is chosen as $x_m$ is at most $k/(c_{3}n)$ for every $m\leqslant M$. Similarly, the probability that $x_m$ is initially infective is at most $X_{\mathrm {I},0}/(c_{3}n)$.

Hence it follows from the comments above, using (2.10) and the assumption $\alpha _nX_{\mathrm {I},0}=O(1)$ in (iii), that $(\zeta _m')_+:=\max (\zeta _m',0)$ has expectation

4.39 $$\begin{aligned} \mathbb {E}{}(\zeta _m')_+ \leqslant \sum _{k=0}^\infty n_k \frac{k}{c_{3}n}\cdot \frac{mk}{c_{3}n}\cdot k +\frac{X_{\mathrm {I},0}}{c_{3}n} =O\left( \frac{m}{n}\right) +O\left( \frac{\alpha _n^{-1}}{n}\right) =O\left( \frac{1}{\alpha _n^2n}\right) =o(\alpha _n). \end{aligned}$$

Let $\bar{Z}_{}':=\sum _{1}^M(\zeta '_m)_+$. Then, by (4.39),

4.40 $$\begin{aligned} \mathbb {E}{}\bar{Z}_{}' = o(M\alpha _n)=o\bigl (\alpha _n^{-1}\bigr ). \end{aligned}$$

Furthermore, for $$m\leqslant M$$,

4.41 $$\begin{aligned} \bar{Z}_{m}^- = \bar{Z}_{m}+\sum _{j=1}^m \zeta '_j \leqslant \bar{Z}_{m}+\bar{Z}_{}'\leqslant \bar{Z}_{m}^++\bar{Z}_{}'. \end{aligned}$$

Since (4.36) and (4.38) hold (in distribution), the sequence $\bigl (\alpha _n\bigl (\bar{Z}_{t\alpha _n^{-2}}^- -Z_0\bigr ) ,\alpha _n\bigl (\bar{Z}_{t\alpha _n^{-2}}^+ -Z_0\bigr )\bigr )$, $n\geqslant 1$, is tight in $D[0,A]\times D[0,A]$. Moreover, every subsequential limit in distribution must be of the form $\bigl (\sigma B^-_t + \lambda _2^{-1}t ,\sigma B^+_t + \lambda _2^{-1}t \bigr )$ for some Brownian motions $B^-_t$ and $B^+_t$. Since $\alpha _n\bar{Z}_{}'\overset{\mathrm {p}}{\longrightarrow }0$ by (4.40), it then follows from (4.41) that for any fixed $t\in [0,A]$, $B_t^-\leqslant B_t^+$ a.s. Since $B_t^-$ and $B_t^+$ have the same distribution, this implies $B_t^-=B_t^+$ a.s. for every fixed *t*, and thus by continuity a.s. for all $t\in [0,A]$.

Since all subsequential limits thus are the same, this shows that (4.36) and (4.38) hold jointly (in distribution) with $B_t^-=B_t$. Finally, by (4.41) and (4.40), this implies

4.42 $$\begin{aligned} \alpha _n\left( \bar{Z}_{t\alpha _n^{-2}} -Z_0\right) \overset{\mathrm {d}}{\longrightarrow }\sigma B_t + \lambda _2^{-1}t , \quad \text {in}\; D[0,A]. \end{aligned}$$

Since the infimum is a continuous functional on *D*[0, *A*], it follows that

4.43 $$\begin{aligned} \alpha _n\left( \inf _{t\leqslant A}\bar{Z}_{t\alpha _n^{-2}} -Z_0\right) \overset{\mathrm {d}}{\longrightarrow }\inf _{t\leqslant A}\bigl ( \sigma B_t + \lambda _2^{-1}t\bigr ) . \end{aligned}$$

For convenience, denote the left- and right-hand sides of (4.43) by $Y_n$ and *Y*. Since the random variable *Y* has a continuous distribution, (4.43) implies that, uniformly in $x\in \mathbb {R}$,

4.44 $$\begin{aligned} \mathbb {P}{}\bigl (Y_n \leqslant x\bigr )=\mathbb {P}{}( Y\leqslant x)+o(1). \end{aligned}$$

The Brownian motion $B_t$ in (4.42) and (4.43) is arbitrary, so we may and shall assume that $B_t$ is independent of everything else.

We have defined $m_*:=\min \{m\geqslant 0:\bar{Z}_{m}=0\}$ and $M:=\lfloor A\alpha _n^{-2}\rfloor$, and thus

4.45 $$\begin{aligned} \mathbb {P}{}\bigl (m_*\leqslant M\bigr ) =\mathbb {P}{}\left( \inf _{t\leqslant A}\bar{Z}_{t\alpha _n^{-2}}\leqslant 0\right) =\mathbb {P}{}\bigl (Y_n\leqslant -\alpha _n Z_0\bigr ). \end{aligned}$$

Recall that $\zeta ^+_m$ and $\zeta ^-_m$ above are independent of $Z_0$. Hence, if we fix two real numbers *a* and *b*, and condition on the event $\mathcal E_n^{a,b}:=\{a\leqslant \alpha _n Z_0 <b\}$, then for every subsequence such that $\liminf _{n\rightarrow \infty }\mathbb {P}{}(\mathcal E_n^{a,b})>0$, the arguments above leading to (4.36), (4.38) and (4.42)–(4.44) still hold. (We need $\liminf _{n\rightarrow \infty }\mathbb {P}{}(\mathcal E_n^{a,b})>0$ in order to get a conditional version of (4.40).) Consequently, $\mathbb {P}{}\bigl (Y_n \leqslant x\mid \mathcal E_n^{a,b}\bigr )=\mathbb {P}{}( Y\leqslant x)+o(1)$, and thus, recalling that $B_t$ is independent of $Z_0$,

4.46 $$\begin{aligned} \mathbb {P}{}\bigl (Y_n \leqslant x\text { and }\mathcal E_n^{a,b}\bigr )=\mathbb {P}{}( Y\leqslant x)\mathbb {P}{}(\mathcal E_n^{a,b})+o(1) =\mathbb {P}{}( Y\leqslant x\text { and }\mathcal E_n^{a,b})+o(1). \end{aligned}$$

On the other hand, (4.46) holds trivially if $\mathbb {P}{}(\mathcal E_n^{a,b})\rightarrow 0$. Every subsequence has a subsubsequence such that either $\liminf _{n\rightarrow \infty }\mathbb {P}{}(\mathcal E_n^{a,b})>0$ or $\mathbb {P}{}(\mathcal E_n^{a,b})\rightarrow 0$, and in any case (4.46) holds along the subsubsequence; it follows that (4.46) holds for the full sequence.

In particular, for any *a* and *b*,

4.47 $$\begin{aligned} \begin{aligned} \mathbb {P}{}\bigl (Y_n\leqslant -\alpha _nZ_0\text { and }\mathcal E_n^{a,b}\bigr )&\leqslant \mathbb {P}{}\bigl (Y_n\leqslant -a\text { and }\mathcal E_n^{a,b}\bigr ) =\mathbb {P}{}\bigl (Y\leqslant -a\text { and }\mathcal E_n^{a,b}\bigr )+o(1)\\&\leqslant \mathbb {P}{}\bigl (Y\leqslant -\alpha _nZ_0+b-a\text { and }\mathcal E_n^{a,b}\bigr )+o(1). \end{aligned} \end{aligned}$$

By assumption, $\alpha _nX_{\mathrm {I},0}$ is bounded, say $\alpha _nX_{\mathrm {I},0}\leqslant C$ for some constant *C*; thus $0\leqslant \alpha _nZ_0\leqslant \alpha _nX_{\mathrm {I},0}\leqslant C$. Let $\delta >0$ and divide the interval [0, *C*] into a finite number of subintervals $[a_j,b_j]$ with lengths $b_j-a_j<\delta$. By summing (4.47) for these intervals, we obtain

4.48 $$\begin{aligned} \begin{aligned} \mathbb {P}{}\bigl (Y_n\leqslant -\alpha _nZ_0\bigr ) \leqslant \mathbb {P}{}\bigl (Y\leqslant -\alpha _nZ_0+\delta \bigr )+o(1). \end{aligned} \end{aligned}$$

Since $\delta >0$ is arbitrary, this implies

4.49 $$\begin{aligned} \begin{aligned} \mathbb {P}{}\bigl (Y_n\leqslant -\alpha _nZ_0\bigr ) \leqslant \mathbb {P}{}\bigl (Y\leqslant -\alpha _nZ_0\bigr )+o(1). \end{aligned} \end{aligned}$$

Similarly, we obtain $\mathbb {P}{}\bigl (Y_n\leqslant -\alpha _nZ_0\bigr ) \geqslant \mathbb {P}{}\bigl (Y\leqslant -\alpha _nZ_0-\delta \bigr )+o(1)$ and $\mathbb {P}{}\bigl (Y_n\leqslant -\alpha _nZ_0\bigr ) \geqslant \mathbb {P}{}\bigl (Y\leqslant -\alpha _nZ_0\bigr )+o(1)$. Consequently,

4.50 $$\begin{aligned} \mathbb {P}{}\bigl (Y_n\leqslant -\alpha _nZ_0\bigr ) = \mathbb {P}{}\bigl (Y\leqslant -\alpha _nZ_0\bigr )+o(1). \end{aligned}$$

In other words, using (4.45) and recalling the meaning of *Y* from (4.43),

4.51 $$\begin{aligned} \begin{aligned} \mathbb {P}{}\bigl (m_*\leqslant M\bigr ) = \mathbb {P}{}\left( \inf _{t\leqslant A}\bigl (\sigma B_t + \lambda _2^{-1}t \bigr )\leqslant -\alpha _n Z_0\right) +o(1). \end{aligned} \end{aligned}$$

If $m_*\leqslant M=\lfloor A\alpha _n^{-2}\rfloor$, then, similarly as (4.37) in the proof of case (i),

4.52 $$\begin{aligned} {\mathcal Z}\leqslant m_*\leqslant A\alpha _n^{-2}=o\bigl (n\alpha _n\bigr ) \end{aligned}$$

so we are in case (a) in Theorem 2.4 (i).

If $$m_*>M$$, consider again $$m_{**}$$ defined by (4.13) (but taking minimum over $$m \geqslant M$$), for a sufficiently small $$\delta >0$$. Note that, as in the proof of (ii), if $$\bar{Z}_{m_{**}}>0$$, then (4.19) holds and w.h.p. $$T^*>\varepsilon \alpha _n$$ for some small $$\varepsilon >0$$, and thus w.h.p. (b) in Theorem 2.4(i) holds. In other words, for some small $$\varepsilon >0$$, if $$m_*\leqslant M$$, then $${\mathcal Z}<\varepsilon n \alpha _n$$, and if $$m_*>M$$ and $$\bar{Z}_{m_{**}}>0$$, then $${\mathcal Z}>\varepsilon n\alpha _n$$ w.h.p.

We next show that the probability that neither of these happens is small. We condition on $$\bar{Z}_{M}$$ and argue as in the proof of case (ii), using Lemma 4.2 on $$\bar{Z}_{(M+m)\wedge m_{**}}-\bar{Z}_{M}$$, and find

4.53 $$\begin{aligned} \mathbb {P}{}\bigl (m_*>M\text { and } \bar{Z}_{m_{**}}=0\mid \bar{Z}_{M}\bigr ) \leqslant \frac{8c_{2}}{c_{1}\alpha _n\bar{Z}_{M}} . \end{aligned}$$

Hence, using also (4.42),

4.54 $$\begin{aligned} \mathbb {P}{}\bigl (m_*>M\text { and } \bar{Z}_{m_{**}}=0\bigr )&\leqslant \mathbb {P}{}(\alpha _n\bar{Z}_{M}< \tfrac{1}{2}\lambda _2^{-1}A) + O(1/A) \nonumber \\&\leqslant \mathbb {P}{}\bigl (\sigma B_A+\lambda _2^{-1}A < \tfrac{1}{2}\lambda _2^{-1}A\bigr )+o(1) + O(1/A) \nonumber \\&=O(1/A)+o(1) . \end{aligned}$$

Using (4.51) and the comments above, it follows that, if $\varepsilon >0$ is small enough, then

4.55 $$\begin{aligned} \mathbb {P}{}({\mathcal Z}<\varepsilon \alpha _nn)&=\mathbb {P}{}\bigl (m_*\leqslant M\bigr )+O(1/A)+o(1) \nonumber \\&= \mathbb {P}{}\left( \inf _{t\leqslant A}\bigl (\sigma B_t + \lambda _2^{-1}t \bigr )\leqslant -\alpha _n Z_0\right) +O(1/A)+o(1). \end{aligned}$$

This holds for every fixed $A>0$, and we can then let $A\rightarrow \infty$ and conclude that

4.56 $$\begin{aligned} \begin{aligned} \mathbb {P}{}({\mathcal Z}<\varepsilon \alpha _nn) = \mathbb {P}{}\left( \inf _{0\leqslant t<\infty }\bigl (\sigma B_t + \lambda _2^{-1}t \bigr )\leqslant -\alpha _n Z_0\right) +o(1). \end{aligned} \end{aligned}$$

It is well-known that $-\inf _{t\geqslant 0}\bigl (\sigma B_t + \lambda _2^{-1}t \bigr )$ has an exponential distribution with parameter $2\lambda _2^{-1}/\sigma ^2$, see e.g. Revuz and Yor (1999, Exercise II.(3.12)). Consequently, since $Z_0$ and $(B_t)$ are independent,

4.57 $$\begin{aligned} \begin{aligned} \mathbb {P}{}({\mathcal Z}<\varepsilon \alpha _nn)&= \mathbb {E}{}\exp \bigl (-2\lambda _2^{-1}\sigma ^{-2}\alpha _nZ_0\bigr ) +o(1). \end{aligned} \end{aligned}$$

Since we assume that $\alpha _nX_{\mathrm {I},0}$ is bounded above and below, $Z_0\leqslant X_{\mathrm {I},0}$ and Lemma 4.1(ii) imply that the expectation in (4.57) stays away from 0 and 1 as ${n\rightarrow \infty }$. Moreover, if $d_{\mathrm {I},*}=o(X_{\mathrm {I},0})$, then Lemma 4.1(i) and (4.57) yield, using (4.33),

4.58 $$\begin{aligned} \mathbb {P}{}({\mathcal Z}<\varepsilon \alpha _nn)&= \exp \bigl (-2\lambda _2^{-1}\sigma ^{-2}\alpha _n\pi _nX_{\mathrm {I},0}\bigr )+o(1)\nonumber \\&= \exp \bigl (-2\lambda \lambda _2^{-2}\sigma ^{-2}\alpha _nX_{\mathrm {I},0}\bigr )+o(1), \end{aligned}$$

which yields (2.18) by the definition of $\sigma ^2$ in (4.34).

Finally, (4.52) and the argument above, in particular (4.54), shows that

4.59 $$\begin{aligned} \mathbb {P}{}\bigl (\text {the epidemic is small but }{\mathcal Z}>A\alpha _n^{-2}\bigr )=O(1/A)+o(1), \end{aligned}$$

which implies the final claim. $\square$

### Proof of Theorem 2.5(iii) in the simple graph case

As said in Sect. 2, this result for the random simple graph $G$ does not follow immediately from the multigraph case (as the other results in this paper do). We use here instead the argument for the corresponding result in Janson et al. (2014, Section 6), with minor modifications as follows. We continue to work with the random multigraph $G^*$. Also, we now allow initially recovered vertices, since our trick in Sect. 3.1 to eliminate them does not work for the simple graph case.

Fix a sequence $\varepsilon _n\rightarrow 0$ such that Theorem 2.4(i) holds, and let ${\mathcal L}$ be the event that there are less than $\varepsilon _n^{1/2}n_{\mathrm {S}}\alpha _n$ pairing events; note that if ${\mathcal L}$ occurs, then ${\mathcal Z}<\varepsilon _n^{1/2}n_{\mathrm {S}}\alpha _n$, while if ${\mathcal L}$ does not occur, w.h.p. ${\mathcal Z}>\varepsilon _nn_{\mathrm {S}}\alpha _n$ by a simple argument (using e.g. Chebyshev’s inequality); hence ${\mathcal L}$ says w.h.p. that the epidemic is small.

Furthermore, let *W* be the number of loops and pairs of parallel edges in $G^*$; thus $G^*$ is simple if and only if $W=0$, and we are interested in the conditional probability $\mathbb {P}{}({\mathcal L}\mid W=0)$. By Janson (2014) (at least if we consider suitable subsequences), $W\overset{\mathrm {d}}{\longrightarrow }\widehat{W}$ for some random variable $\widehat{W}$, with convergence of all moments.

We write $W=W_1+W_2$, where $W_2$ is the number of loops and pairs of parallel edges that include either an initially infective vertex (as in Janson et al. 2014), or a vertex with degree at least $\overline{d}:=1/\alpha _n$. Then, by the assumptions

4.60 $$\begin{aligned} \mathbb {E}{}W_2 = O\left( \left( \sum _{k=0}^\infty k^2 n_{\mathrm {I},k}+ \sum _{k\geqslant \overline{d}}k^2(n_{\mathrm {S},k}+n_{\mathrm {R},k}) \right) \left( \frac{1}{n}+\frac{\sum _{k=0}^\infty k^2n_{k}}{n^2}\right) \right) =o(1) \end{aligned}$$

and thus it suffices to consider $W_1$. Note also that if we fix a vertex *v* that is not initially infected and has degree less than $\overline{d}$, then the probability that the infection will reach *v* within less than $\varepsilon _n^{1/2}n_{\mathrm {S}}\alpha _n$ pairing events is $O\bigl (\overline{d}\varepsilon _n^{1/2}n \alpha _n/n\bigr )=o(1)$, so w.h.p. *v* is not infected before it is determined whether ${\mathcal L}$ occurs or not.

The rest of the proof is exactly as in Janson et al. (2014), to which we refer for details. $\square$

### Remark 4.4

The formula (2.18) for the asymptotic probability that the epidemic is small holds only under the assumption $d_{\mathrm {I},*}=o(X_{\mathrm {I},0})$, i.e., that among the initially infective vertices, no vertex has a significant fraction of all their half-edges. Even if this assumption does not hold, the asymptotic probability can be found from (4.57), since as in the proof of Lemma 4.1, $Z_0=\sum _i Z_{0,i}$ where the $Z_{0,i}$ are independent and, using the notation in Lemma 4.3, $Z_{0,i}\overset{\mathrm {d}}{=}Y(d_{\mathrm {I},i}+1)$, where $d_{\mathrm {I},i}$ is the degree of the *i*-th initially infective vertex. Hence, letting $\chi$ denote the fraction in (2.18), so $\chi \sim 2\lambda _2^{-1}\sigma ^{-2}\pi _n$, the probability is

4.61 $$\begin{aligned} \begin{aligned}&\prod _i\mathbb {E}{}\exp \bigl (-\chi \pi _n^{-1}\alpha _nY(d_{\mathrm {I},i}+1)\bigr )+o(1)\\&\qquad =\prod _{k}\bigl (\mathbb {E}{}\exp \bigl (-\chi \pi _n^{-1}\alpha _nY(k+1)\bigr )\bigr )^{n_{\mathrm {I},k}}+o(1). \end{aligned} \end{aligned}$$

A calculation, see Appendix C, shows that if we define

4.62 $$\begin{aligned} \psi _n(k):= \log \int _0^1\exp \left( k\alpha _n\chi \pi _n^{-1}\left( x^{\beta _n/\rho _n}-\tfrac{\rho _n}{\beta _n+\rho _n}\right) \right) \,dx, \end{aligned}$$

interpreted as 0 when $\rho _n=0$, then this probability is

4.63 $$\begin{aligned} \exp \left( -\chi \alpha _nX_{\mathrm {I},0}+\sum _k n_{\mathrm {I},k}\psi _n(k) \right) +o(1), \end{aligned}$$

thus generalizing (2.18). It is easily seen that $\psi _n(k)=O\bigl (k^2\alpha _n^2\bigr )$ and thus $\sum _k n_{\mathrm {I},k}\psi _n(k)=O\bigl (\alpha _nd_{\mathrm {I},*}\sum _k n_{\mathrm {I},k}k \alpha _n\bigr ) =O\bigl (\alpha _nd_{\mathrm {I},*}\bigr )$ under our assumption $\alpha _nX_{\mathrm {I},0}=O(1)$, which explains why the extra term in (4.63) disappears in (2.18).

Note also that $\psi _n(k)\geqslant 0$ by Jensen’s inequality; thus an extremely uneven distribution of the degrees of the initially infective vertices will increase the probability of a small outbreak.

## Appendix A: The Sellke construction for network epidemics

 House et al. (2012) introduced a method to simulate the final size of a network epidemic that drew on the constructions of  Sellke (1983) and  Ludwig (1975) (the latter in its most general sense as described by  Pellis et al. (2008)). This approach allows us to make a realisation by drawing three sets of random numbers, and then we can consider multiple initial conditions and the final sizes they produce without drawing further random numbers.

Let individuals be labelled $i,j,\ldots \in \{ 1, \ldots , n\}$. Let them be connected by a general network with adjacency matrix with elements $G_{ij}$, and let $Z_i$ be an indicator variable taking the value 1 if individual *i* is eventually infected during the epidemic and 0 otherwise. Our algorithm then proceeds as follows.

First, for each individual *i* pick an infectious period $T_i \sim \mathrm {Exp}(\rho )$. Secondly, for each individual *i* pick a threshold $Q_i\sim \mathrm {Exp}(1)$. This represents the individual’s resistance to infection. Thirdly, a random permulation *P* of the integers $\{1,\ldots ,n\}$ is chosen. The first *m* elements of this permutation are then taken to be the indices of the initially infectious individuals, i.e. we initialise $Z_i\leftarrow 1$ if $i\in P$ and $Z_i\leftarrow 0$ otherwise. Then to arrive at the correct final values we iteratively search for each individual *i* that has $Z_i=0$ and set $Z_i\leftarrow 1$ if

A.1 $$\begin{aligned} Q_i < \beta \sum _j G_{ij} T_j Z_j. \end{aligned}$$

This procedure is continued until no changes occur on a given iteration, giving the final size for that value of *m*. We can then increase *m* and continue the iterative procedure, allowing simulations that are both computationally efficient, and for which the total final size $\mathcal {Z}$ is monotone non-decreasing in the initial number infected *m*.

## Appendix B: Critical behaviour of $G(n, (d_i)_{i=1}^n)$

In this appendix, we show how the methods in this paper yield an improvement of Janson and Luczak (2009, Theorem 2.4) on the size of the largest component in $G(n, (d_i)_{i=1}^n)$ near criticality, replacing the moment condition used in Janson and Luczak (2009)

B.1 $$\begin{aligned} \sum _{k=0}^\infty k^{4+\eta } n_{k}= O(n) \end{aligned}$$

for some $\eta > 0$, by the uniform summability of $\sum _{k=0}^\infty k^{3} n_{k}$ in assumption (ii) below, cf. (D2). This is more or less best possible, since if the limiting vertex degree distribution does not have a finite third moment, then (at least in typical cases), the size of the largest component is $o_{\mathrm p}(n\alpha _n)$, see van der Hofstad, Janson and Luczak (in preparation).

### Theorem B.1

Suppose that the degree sequences $(d_i)_{i=1}^n$ satisfy the following conditions.

(i) There is a probability distribution $(p_k)_{k = 0}^\infty$ such that $n_{k}/n \rightarrow p_k$ for each $k \geqslant 0$.
(ii) For all $\varepsilon > 0$, there exists *M* such that, for all *n*, $\sum _{k=M}^\infty k^3 n_{k}< \varepsilon n$.
(iii) $$p_1 > 0$$.
(iv) $$\sum _{k=0}^\infty k(k-2)p_k = 0$$.
(v) $$\alpha _n {:=} \sum _{k=0}^\infty k(k-2)n_{k}/n$$ satisfies $$n^{1/3} \alpha _n \rightarrow \infty $$.

Define the positive constants $\lambda {:=} \sum _{k=0}^\infty k p_k$ and $\gamma {:=} \sum _{k=0}^\infty k(k-1)(k-2)p_k$.

Let $\mathcal C_1$ and $\mathcal C_2$ denote the largest and second largest components of $G(n, (d_i)_{i = 1}^n)$. Then the number of vertices in $\mathcal C_1$ is

B.2 $$\begin{aligned} v(\mathcal C_1) = \frac{2\lambda }{\gamma }n\alpha _n + o_{\mathrm p}(n\alpha _n), \end{aligned}$$

the number of vertices in $\mathcal C_1$ with degree $k \geqslant 0$ is

B.3 $$\begin{aligned} v_k(\mathcal C_1) = \frac{2k p_k}{\gamma }n\alpha _n + o_{\mathrm p}(n\alpha _n), \end{aligned}$$

and the number of edges in $\mathcal C_1$ is

B.4 $$\begin{aligned} e(\mathcal C_1) = \frac{2\lambda }{\gamma }n\alpha _n + o_{\mathrm p}(n\alpha _n). \end{aligned}$$

In contrast, $\mathcal C_2$ has only $v(\mathcal C_2) = o_{\mathrm p}(n\alpha _n)$ vertices and $e(\mathcal C_2) = o_{\mathrm p}(n\alpha _n)$ edges.

### Remark B.2

If $\sum _k k (k-2) p_k = 0$ then $p_1 > 0$ is equivalent to $p_0+p_1 +p_2 < 1$ (condition (D6)). As a consequence, $\gamma >0$.

### Sketch of proof

We will explain how to modify the argument of Janson and Luczak (2009). The latter is similar in spirit (and actually inspired) our proof of Theorem 2.4 in Sect. 3. Indeed, the algorithm used in Janson and Luczak (2009) to construct the multigraph $G^*(n, (d_i)_{i=1}^n)$ and explore its components is closely related to the time-changed epidemic with zero recovery rate, i.e. $\rho _n = 0$. In more detail, we begin with all vertices susceptible (or *sleeping* in the terminology of Janson and Luczak 2009). We choose a single vertex at random and declare it infective (or *active* in Janson and Luczak 2009). The infection eventually spreads through the whole connected component containing the chosen vertex because $\rho _n = 0$. The connected component thus comprises the susceptible (sleeping) vertices that were infected (activated) during this time, together with the initially infective (active) vertex. The procedure can be repeated until all the connected components have been explored.

In particular, each susceptible (sleeping) vertex of degree $k \geqslant 0$ is still infected (activated) at rate *k* in the algorithm of Janson and Luczak (2009); in addition one vertex is activated at the start of each new component. The number of vertices that would be sleeping if we ignore the latter type of activations (denoted $\tilde{V}_k(t)$ in Janson and Luczak 2009) thus evolves as the Markov death chain $\tilde{S}_{t}(k)$ considered in the proof of Theorem 3.1 but with $\tilde{S}_{0}(k) = n_{k}$. One can prove concentration of measure for $\tilde{S}_{t}(k)$, $\sum _{k=0}^\infty \tilde{S}_{t}(k)$ and $\sum _{k=0}^\infty k \tilde{S}_{t}(k)$, as in Theorem 3.1. The analogous result in Janson and Luczak (2009) is Lemma 6.3 and its proof involves a non-trivial use of assumption (B.1). So we must replace (Janson and Luczak 2009, Lemma 6.3) with Theorem 3.1. Note that the former result achieves an $O_{\mathrm p}(n^{1/2}\alpha _n^{1/2}+n\alpha _n^3)$ bound on the error, versus the $o_{\mathrm p}(n\alpha _n^2)$ bound of Theorem 3.1. However, it can be checked that the $o_{\mathrm p}(n\alpha _n^2)$ bound is sufficient for the rest of the proof.

There is only one other place where Janson and Luczak (2009) uses assumption (B.1) non-trivially; that is to control the Taylor expansion remainder term in the analogue of (3.51) (immediately preceding equation (6.7) on page 212). But we may obtain an adequate bound with the argument leading to (3.49).

All of the other modifications are trivial. $\square$

## Appendix C: Proof of (4.63)

Let $c_n:=\chi \pi _n^{-1}$ and note that $c_n=O(1)$ by (4.1) and (2.20). If $\rho _n>0$, then by the definition of $Y_n(k)$ before Lemma 4.3, and the substitution $x=e^{-\rho _n\tau }$,

C.1 $$\begin{aligned} \mathbb {E}{}e^{-c_n\alpha _n Y(k+1)}&=\int _0^\infty \Bigl (1+\bigl (1-e^{-\beta _n\tau }\bigr )\bigl (e^{-c_n\alpha _n}-1\bigr )\Bigr )^k \rho _n e^{-\rho _n\tau }\,d\tau \nonumber \\&=\int _0^1\Bigl (1-\bigl (1-x^{\beta _n/\rho _n}\bigr )\bigl (1-e^{-c_n\alpha _n}\bigr )\Bigr )^k \,dx \nonumber \\&=\int _0^1\Bigl (1-\bigl (1-x^{\beta _n/\rho _n}\bigr )\bigl (c_n\alpha _n(1+O(\alpha _n))\bigr )\Bigr )^k \,dx\nonumber \\&=\int _0^1\exp \Bigl (-k\bigl (1-x^{\beta _n/\rho _n}\bigr )\bigl (c_n\alpha _n+O(\alpha _n^2)\bigr )\Bigr ) \,dx \nonumber \\&=e^{O(k\alpha _n^2)} \int _0^1\exp \Bigl (c_n\alpha _nk\bigl (x^{\beta _n/\rho _n}-1\bigr )\Bigr ) \,dx \nonumber \\&=e^{-c_n\pi _n\alpha _nk+O(k\alpha _n^2)} \int _0^1\exp \Bigl (c_n\alpha _nk\bigl (x^{\beta _n/\rho _n}-\tfrac{\rho _n}{\beta _n+\rho _n}\bigr )\Bigr ) \,dx\nonumber \\&=e^{-\chi \alpha _nk+\psi _n(k)+O(k\alpha _n^2)}. \end{aligned}$$

If $\rho _n=0$, then $\pi _n=1$ and $Y(k+1)=k$, and (C.1) is trivial.

We obtain (4.63) by using (C.1) in (4.61).
